# Disclosing the essentiality of ribose-5-phosphate isomerase B in Trypanosomatids

**DOI:** 10.1038/srep26937

**Published:** 2016-05-27

**Authors:** Joana Faria, Inês Loureiro, Nuno Santarém, Pedro Cecílio, Sandra Macedo-Ribeiro, Joana Tavares, Anabela Cordeiro-da-Silva

**Affiliations:** 1 Parasite Disease Group, Instituto de Biologia Molecular e Celular da Universidade do Porto, Portugal; 2 Instituto de Investigação e Inovação em Saúde, Universidade do Porto, Porto, Portugal; 3 Protein Crystallography Group, Instituto de Biologia Molecular e Celular da Universidade do Porto, Portugal; 4 Departamento de Ciências Biológicas, Faculdade de Farmácia, Universidade do Porto, Portugal

## Abstract

Ribose-5-phosphate isomerase (RPI) belongs to the non-oxidative branch of the pentose phosphate pathway, catalysing the inter-conversion of D-ribose-5-phosphate and D-ribulose-5-phosphate. Trypanosomatids encode a type B RPI, whereas humans have a structurally unrelated type A, making RPIB worthy of exploration as a potential drug target. Null mutant generation in *Leishmania infantum* was only possible when an episomal copy of *RPIB* gene was provided, and the latter was retained both *in vitro* and *in vivo* in the absence of drug pressure. This suggests the gene is essential for parasite survival. Importantly, the inability to remove the second allele of *RPIB* gene in sKO mutants complemented with an episomal copy of *RPIB* carrying a mutation that abolishes isomerase activity suggests the essentiality is due to its metabolic function. *In vitro*, sKO promastigotes exhibited no defect in growth, metacyclogenesis or macrophage infection, however, an impairment in intracellular amastigotes’ replication was observed. Additionally, mice infected with sKO mutants rescued by RPIB complementation had a reduced parasite burden in the liver. Likewise, *Trypanosoma brucei* is resistant to complete *RPIB* gene removal and mice infected with sKO mutants showed prolonged survival upon infection. Taken together our results genetically validate RPIB as a potential drug target in trypanosomatids.

Some clinically relevant pathogens can survive and replicate in macrophages (MØ). However, only the protozoan *Leishmania* and *Coxiella* bacteria are known to thrive in fully mature phagolysosomes^1^,^2^. Mammals become infected with *Leishmania* through the bite of an infected sand fly, which injects non-replicative metacyclic promastigotes into the skin that are later phagocytosed by MØ^2^. Upon delivery to the phagolysosome, the internalized promastigotes differentiate into amastigotes^3^. The latter are able to rapidly re-infect other phagocytic cells (MØ or dendritic cells), as well as some non-phagocytic cells (fibroblasts), leading to several possible disease outcomes, such as acute disease (ranging from self-healing cutaneous infections to fatal, if untreated, visceral forms), as well as chronic or latent infections^3^.

Leishmaniasis is an important worldwide human health problem, affecting approximately 12 million people, with 1.3 million new cases every year^4^. Visceral leishmaniasis (VL), the most severe form of the disease, is mainly associated with *Leishmania donovani* or *infantum* infections. Currently, control of VL relies mainly on chemotherapy and vector control, both presenting several limitations that hinder disease eradication in endemic areas^5^. Traditional chemotherapy is often associated with high cost, toxicity, complex administration regimes and the emergence of resistance^5^. Consequently, approximately 20,000 to 30,000 people die every year^4^, rendering the search for novel chemotherapeutic options a priority.

The Pentose Phosphate Pathway (PPP) is a key metabolic pathway that relies on the use of glucose and is classically divided into two branches: an oxidative and a non-oxidative branch. In the trypanosomatids, enzymes from the oxidative branch, namely glucose-6-phosphate dehydrogenase (G6PD), 6-phosphogluconolactonase (6PGL) and 6-phosphogluconate dehydrogenase (6PGDH) play an important housekeeping role and are related to their cyanobacteria and plant orthologues^6^,^7^. The non-oxidative PPP is responsible for the interconversion of phosphorylated saccharides, giving rise to products (ribose-5-phosphate - R5P), intermediates (glyceraldehyde-3-phosphate - G3P, fructose-6-phosphate - F6P) and cofactors (NADPH) used to synthesize nucleic acids and lipids and to maintain redox homeostasis^8^. Curiously, the enzymes involved in the non-oxidative branch, such as ribose-5-phosphate isomerase B (RPIB), ribose-5-phosphate-3-epimerase (RPE), transketolase (TKT) and transaldolase (TAL), constitute a more heterogeneous group, including a member that lacks any mammalian orthologue (RPIB^9^) and others (RPE and TKT) that are developmentally regulated and dispensable in a species-specific manner^10^,^11^,^12^,^13^,^14^.

RPI enzymes catalyse the interconversion of ribose 5-phosphate (R5P) and ribulose 5-phosphate (Ru5P), depending on the substrate and product concentrations^15^. Two types of RPI enzymes can be found: type A (RPIA) is represented in all plant and animal kingdoms, contrasting with the type B (RPIB) that is restricted to some bacteria and protozoans^15^. Trypanosomatids possess a RPI type B which has no mammalian homologue^9^,^16^,^17^,^18^. Indeed, we have recently demonstrated that in *Trypanosoma brucei* bloodstream forms, RPIB knockdown induces a dramatic impairment in parasite infectivity^18^.

Taken together, RPIB appeared suitable for investigation as a drug target candidate in *Leishmania*. Thus, to evaluate the importance of this protein for *Leishmania* survival and infectivity, we have performed target gene replacement studies in *L. infantum*, and characterized the mutants generated. Target gene replacement studies were also undertaken in *T. brucei.*

## Results

### *Li*RPIB and *Lm*RPIB sequence alignment

**Figure 1:**
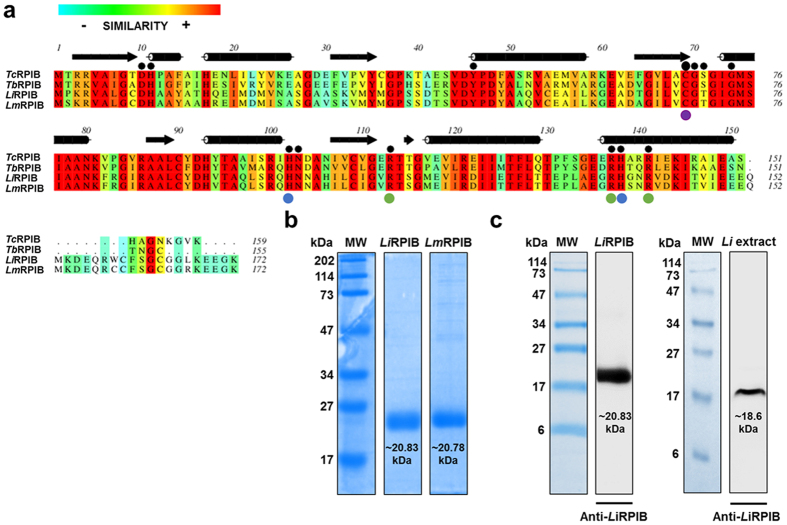
Multiple-sequence alignment of RPIB proteins from trypanosomatids and analysis of recombinant *Li*RPIB and *Lm*RPIB. (**a)** Alignment of *Li*RPIB (NCBI-Gene ID: 5070424/LinJ.28.2100), *Lm*RPIB (NCBI-Gene ID: 5653408/LmjF.28.1970), *Tb*RPIB (NCBI-Gene ID: 3664062/Tb927.11.8970) and *Tc*RPIB (NCBI-Gene ID: 3542840/TcCLB.508601.119). The colour pattern according to ALSCRIPT Calcons (Aline version 011208) indicates: red, identical residues; orange to blue, scale of conservation of amino acid properties in each column alignment; white, dissimilar residues). Secondary structure components of *Tc*RPIB crystal structure (black) are represented above the alignment (arrows correspond to beta-sheets and cylinders correspond to alpha-helices). In all sequences, residues involved in ligand binding, isomerisation and the architecture of the catalytic pocket were represented with black circles above the alignment: Purple, light green and light blue circles below the alignment indicate the residues involved in the isomerisation, ring opening and phosphate stabilisation, respectively. **(b)** Coomassie blue stained SDS-PAGE gel of 10 μg of recombinant *Li*RPIB and *Lm*RPIB post affinity chromatography purification. (**c)** Western-blot analysis of recombinant *Li*RPIB or whole *L. infantum* promastigote extract using a rabbit polyclonal anti-*Li*RPIB (1:1000). MW, molecular weight marker.

The open reading frames (ORFs) encoding a putative RPIB enzyme were identified in the genomes of *L. infantum* JPCM5 (LinJ.28.2100) and *L. major* Friedlin (LmjF.28.1970)^19^,^20^. The *RPIB* amplified sequences from these strains matched 100% with the annotated sequences. RPIB sequences of *L. infantum (Li*RPIB), *L. major* (*Lm*RPIB), *T. brucei* (*Tb*RPIB) and *T. cruzi* (*Tc*RPIB) generate polypeptides containing 172, 172, 155 and 159 amino acids, respectively (Fig. 1a). *Li*RPIB displays a 93% sequence identity with *Lm*RPIB, and around 50% to RPIB from trypanosomes. However, *Li*RPIB possesses s only approximately 12% similarity to human RPIA. The amino acids involved in isomerisation (Cys69), phosphate stabilization (Arg113, Arg137, Arg141) and ring opening (His102, His138) have been identified in *Tc*RPIB^16^ and are strictly conserved in the *Li*RPIB, *Lm*RPIB and *Tb*RPIB sequences (Fig. 1a).

### Enzymatic characterization of *Li*RPIB and *Lm*RPIB

*Li*RPIB and *Lm*RPIB recombinant proteins, containing a 6-histidine N-terminal tag, were expressed in *Escherichia coli* and purified by affinity chromatography under native conditions. Coomassie blue is represented in Fig. 1b, showing that both proteins have the predicted MW for the monomer (*Li*RPIB ~20.83, *Lm*RPIB ~20.78 kDa). Coomassie staining also demonstrates that the purified recombinant proteins possess a suitable purity level to proceed to enzymatic studies. Additionally, recombinant *Li*RPIB was used to immunise rabbits to produce polyclonal anti-*Li*RPIB antibodies. Those antibodies recognise the recombinant protein and a single band in an extract of *L. infantum* promastigotes with the expected MW (~18.6 kDa, [web.expasy.org/protparam]) (Fig. 1c). Both *Li*RPIB and *Lm*RPIB catalyse the conversion of R5P into Ru5P (direct reaction) or Ru5P into R5P (reverse reaction) *in vitro*, with similar kinetic constants, as shown in Supplementary Table S1. The reverse reaction, which generates R5P, seems to be favoured, with both enzymes presenting lower *K*_m_ values and higher *v*_max_ and *k*_cat_.

### RPIB expression and localisation in *L. infantum* promastigote and amastigote forms

The rabbit anti-*Li*RPIB polyclonal antibody was also validated by IFA, as the fluorescence intensity of WT promastigotes *versus LiRPIB* mutants (single knockout (sKO; clone 15) and overexpressing line (OE)) was measured and a positive correlation between protein levels and labelling intensity was found (Supplementary Fig. S1).

**Figure 2:**
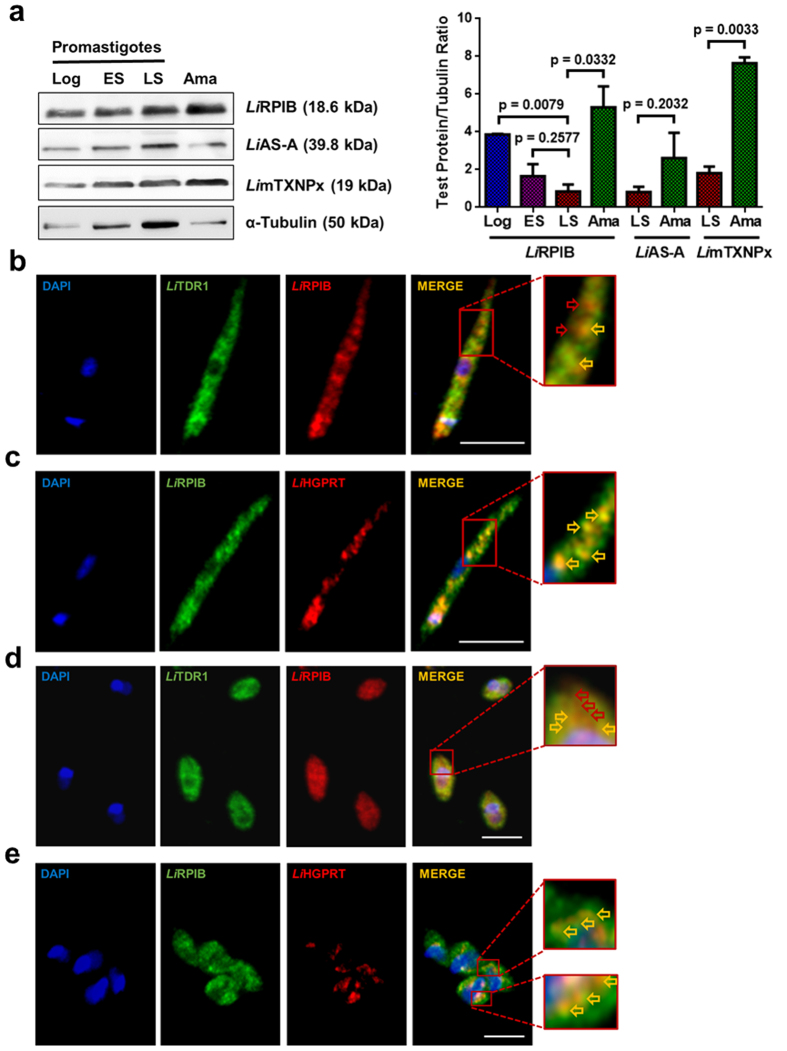
RPIB expression and localization in *L. infantum*. **(a)** RPIB expression in different stages of the *L. infantum* life cycle. Promastigote forms: logarithmic phase (Log), early stationary phase (ES), late stationary phase (LS); axenic amastigote forms (Ama). Twenty μg of total extracts were analysed by Western-blot and probed with rabbit polyclonal anti-*Li*RPIB, anti-*Li*AS-A or *Li*mTXNPx. α-tubulin, probed using a mouse monoclonal antibodywas used as loading control. Quantification, expressed in test protein/tubulin ratio, is presented in the right panel. Two-tailed unpaired *t-*test was performed: statistical significance p < 0.05. **(b–e)** Immunofluorescence analysis showing *Li*RPIB (red in **b** and **d**; green in **c** and **e**) localization in *L. infantum* promastigote (**b,c**) and amastigote (**d,e**) forms. Nucleus and kinetoplast DNA, cytosol and glycosomes were stained with DAPI (blue), sheep anti-*Li*TDR1 (thiol-dependent reductase 1, green) and rabbit anti-*Li*HGPRT (red), respectively. On panels **b** and **d**, red arrows in the zoomed areas correspond to sites of exclusive *Li*RPIB staining. On panels **b–e**, yellow arrows in the zoomed areas point to colocalisation sites. Images are maximal Z-projections of 30 to 35 contiguous stacks separated by 0.1 μm and were acquired with a 63x objective, using a LEICA SP5II confocal microscope. The scale bar corresponds to 5 μm. Data displayed in **a–e** are representative of three independent experiments.

Using α-tubulin as a loading control and Western-blot analysis, we compared RPIB expression in different stages of the parasite’s life cycle. RPIB was found to be more highly expressed in amastigotes (5 fold increase) followed by logarithmic promastigotes (3 fold increase) when compared to late stationary phase promastigotes (Fig. 2a). *Li*AS-A (asparagine synthetase A^21^), which is equally expressed in the different stages, and *Li*mTXNPx (mitochondrial tryparedoxin^22^), which is more expressed in amastigotes, were used as controls (Fig. 2a). This result was also supported by immunofluorescence analysis, as the intensity of the RPIB signal, normalized for the parasite area, was higher in amastigotes, whereas in the case of AS-A no significant difference was observed (Supplementary Fig. S2).

To determine *Li*RPIB subcellular localization, we resorted to *in silico* prediction and performed digitonin fractionation and immunofluorescence analysis. Bioinformatics tools, Wolf Psort and CELLO, predicted a cytosolic localization for RPIB. Digitonin fractionation followed by Western-blot analysis was performed in promastigotes. The following antibodies were used to detect the fractioning pattern of proteins located in different subcellular compartments, namely, anti-*Tb*Enolase (*Li*Enolase *versus Tb*Enolase 79% identity, *Li*Enolase 39.6 kDa) as a cytosolic marker^23^ and anti-*Ld*HGPRT (hypoxanthine guanine phosphoribosyl transferase, *Li*HGPRT 23.6 kDa) as a glycosomal marker^24^. Enolase (Supplementary Fig. S3a) was found in the supernatant at digitonin concentrations as low as 12.5 μg/ml. HGPRT, in turn, was retained longer in the pellet fractions (detected up to 1000 μg/ml), being significantly released in the supernatant only at 100 μg/ml. RPIB is released in the supernatant and retained in the pellet fractions similarly to Enolase and HGPRT, respectively. To further elucidate RPIB localization, we have also performed immunofluorescence in promastigotes and axenic amastigotes, followed by confocal microscopy. TDR1 (thiol-dependent reductase 1)^25^ and HGPRT were labelled as cytosolic and glycosomal markers, respectively. Representative images of the labelling with these two markers are shown in Supplementary Figs S3b and S4a. The RPIB distribution pattern and the co-localization with the cytosolic marker TDR1, with exception of few areas (highlighted with red arrows in Fig. 2b,d, and Supplementary Figs S3c and S4b), indicate that the protein is mainly localized in the cytosol. However, some co-localization with the glycosomal marker, HGPRT, was observed (Fig. 2c,e, and Supplementary Figs S3d and S4c), suggesting this protein might have a dual localization. Indeed, in all the parasites analysed, we always observed partial co-localization of RPIB with TDR1 and HGPRT. The images provided are representative of these observations.

In conclusion, RPIB despite having mainly cytosolic localisation in both promastigotes and axenic amastigotes can also be found in association with glycosomes.

### *LiRPIB* facilitated null mutant generation by targeted gene replacement

A targeted gene replacement strategy was used for *RPIB* gene inactivation in *L. infantum*. The first transfection allowed the removal of one of the gene alleles generating sKO clones resistant to either neomycin *(NEO)* or hygromycin *(HYG),* depending on the cassette that was used. When attempting to remove the second gene allele, independently of using a *HYG* or *NEO*, no null mutants were obtained from a total of four independent experiments. Some of the mutants obtained following the dKO attempt were likely to have been aneuploid (Supplementary Table S2), as the parasites were shown by PCR and Southern-blot to carry both resistance cassettes and the gene at the target locus (data not shown). The repeated inability to remove the second allele, coupled with the constant aneuploidy generation was suggestive of gene essentiality. Therefore, a *HYG* construct has been transfected in parallel into parasites with the following genotypes: WT, sKO*NEO* (sKO 15) and sKO*NEO* complemented (sKO P15) with an episome carrying the *RPIB* gene (pSP*αBLASTαRPIB*). This approach enabled the generation of sKO mutants from the WT and dKO mutants only in the complemented sKO, therefore once more failing to generate mutants that were truly null in terms of *RPIB*. The WT parasites were transfected with the pSP*αBLASTαRPIB* plasmid to generate an overexpressing cell-line.

**Figure 3:**
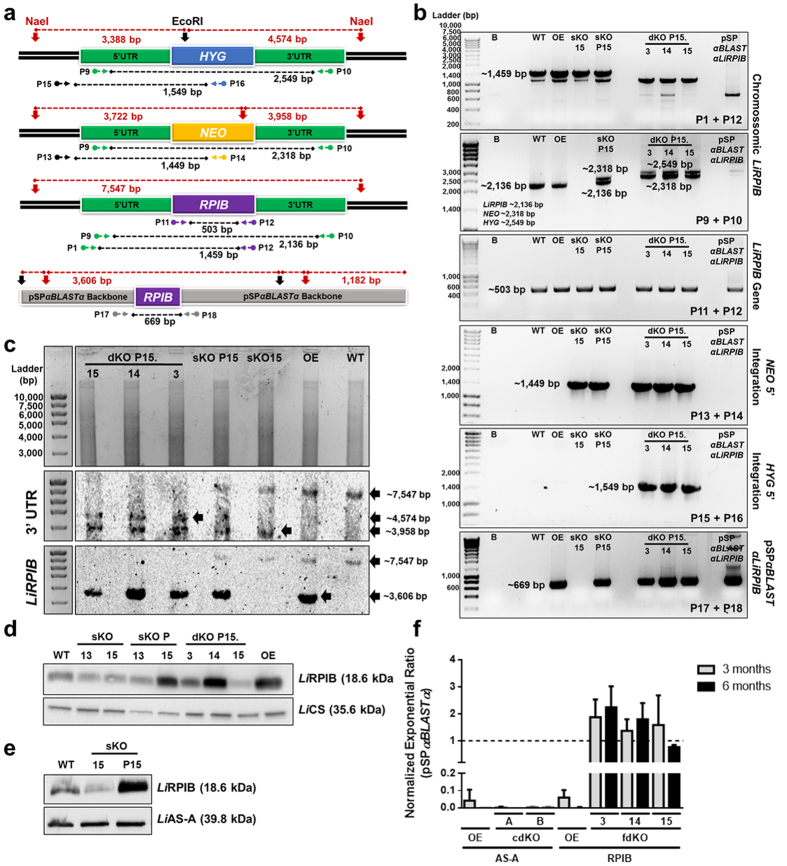
Genetic and post-translational analysis of the *LiRPIB* mutants. **(a)** *LiRPIB* locus (*RPIB* allele, targeted gene replacement cassettes, containing *NEO* and *HYG*) and pSP*αBLASTαLiRPIB* schematics. Horizontal arrows and numbers represent the primer pairs (sequences in Table S4) used to assess the genotype of the mutants: the black dashed line represents the expected PCR fragment. The colour of those arrows indicates the region of annealing: *HYG*, *NEO*, *RPIB*, UTRs used in the replacement cassettes, endogenous regions of the parasite or pSP*αBLASTαLiRPIB* backbone in blue, yellow, purple, green, black or grey, respectively. Southern-blot approach, upon digestion with *EcoR*I (vertical black arrows) and *Nae*I (vertical red arrows) is also represented: dashed red lines represent the expected digestion fragments. **(b)** PCR analysis of *LiRPIB* mutants to assess *LiRPIB, NEO* and *HYG* presence in chromosome 28, as well as pSP*αBLASTαLiRPIB* presence. B, blank. **(c)** Southern-blot analysis of 10 μg of *LiRPIB* mutants (*versus* WT) genomic DNA, previously digested with *EcoR*I and *Nae*I, and probed using 3′ UTR. The blot was then stripped and reprobed using *LiRPIB*. The horizontal arrows highlight the bands that were obtained. **(d,e)** Western-blot analysis of RPIB expression in the *LiRPIB* mutants (*versus* WT), in both promastigotes (**d**) and axenic amastigotes (**e**). 1 × 10^7^ parasites were used for total extract and *Li*CS (cysteine synthase) or *Li*AS-A (asparagine synthetase A) were used as loading control in d and e, respectively. **(f)** pSP*αBLASTα* quantification by qPCR using 10 ng of genomic DNA from *LiRPIB* OE and facilitated null mutants (fdKO) P15.3, P15.14 and P15.15 and from *LiASA* OE and complemented null mutants (cdKO) clones A and B. We used DNA from each of the mutants cultured for 3 and 6 months in complete RPMI in the absence of blasticidin and calibrated against the correspondent mutant maintained in culture in the presence of the drug. rRNA45 was used as the reference gene. The results correspond to means plus standard deviation of two independent experiments. WT, wild type; OE, overexpressor; sKO, single knockout (clones 13 and 15); sKO P, sKO (clones 13 and 15) complemented with pSP*αBLASTαLiRPIB;* dKO P15, facilitated null mutants (clones 3, 14 and 15).

The genotype of the facilitated null mutants (dKO P15.3, dKO P15.14 and dKO P15.15) was confirmed by PCR (Fig. 3b) and Southern-blot (Fig. 3c), these respective strategies are illustrated in Fig. 3a. The successful integration of the selectable markers, the presence of *RPIB* in the episome and its absence from the chromosomic locus were verified by PCR. The genotypes were further confirmed by Southern-blot. A first hybridization was performed using 3′ UTR as a probe: the bands of ~7547 bp, ~3958 bp and ~4574 bp correspond to *RPIB, NEO* and *HYG,* respectively (Fig. 3c). The blot was then stripped and re-probed three additional times to confirm each of the bands. Re-probing with *RPIB* gene confirmed its presence in the chromosomes of WT, OE, sKO 15 and sKO P15 (~7547 bp) and in the episome in OE, sKO P15 and facilitated null mutants (~3606 bp) (Fig. 3c). All the clones in the promastigote stage were also analysed by Western-blot and showed RPIB reduction in sKO and overexpression in the sKO P15 and OE mutants (Fig. 3d). As expected, RPIB levels are also down regulated in the axenic amastigotes sKO (clone 15) when compared to WT or to the complemented line (sKO P15) (Fig. 3e).

The facilitated dKO mutants were subcultured weekly for 6 months in the presence or absence of blasticidin drug pressure. In the absence of drug pressure, *LiRPIB* facilitated mutants (fdKO) did not lose the plasmid, as assessed by qPCR (Fig. 3f). On the other hand, *Li*RPIB OE, as well as an unrelated non-essential protein (*Li*AS-A^21^) OE and its complemented null mutants (also using pSP*αBLASTα* as a vector; cdKO), lost the plasmid. Importantly, *LiRPIB* facilitated null mutant promastigotes when cultured in RPMI or SDM enriched or not with D-ribose do not lose the plasmid suggesting D-ribose supplementation appears not to compensate for the need of RPIB (Supplementary Fig. S5).

In conclusion, our results indicate RPIB is essential for the survival of *L. infantum*.

### Impact of *LiRPIB* partial inactivation on *in vitro* growth and infection

Several experiments using the different mutants were conducted *in vitro* to assess whether *Li*RPIB has a role in promastigote growth and metacyclogenesis, MØ infection and/or intracellular amastigote replication.

**Figure 4:**
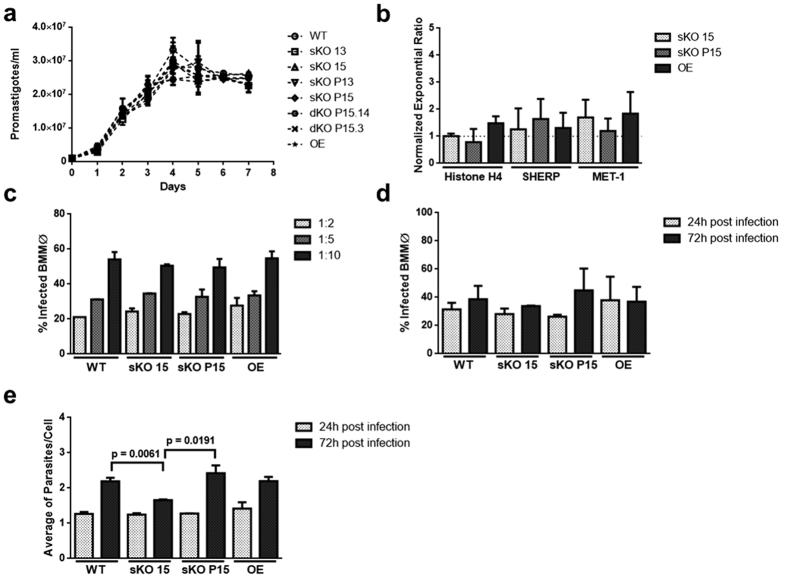
*In vitro* characterization of *LiRPIB* mutants: promastigotes growth, metacyclogenesis, MØ invasion and intracellular amastigote replication. (**a**) *L. infantum* promastigotes growth curves of *LiRPIB* mutants (*versus* WT), cultured in cRPMI. Data correspond to mean values of duplicates ± standard deviation. (**b**) Histone H4, SHERP and MET-1 gene quantification of expression levels by qRT-PCR. Total RNA from WT and *LiRPIB* mutant stationary promastigotes was extracted and converted into cDNA. rRNA45 was used as reference gene. Calibration was performed against WT parasite. Data correspond to mean values of duplicates plus standard deviation. (**c**) Percentage of infected bone marrow derived MØ at 4 hours post infection, determined by FACS analysis using CFSE-labelled WT or *LiRPIB* mutant stationary promastigotes. Infections were performed using a ratio of 2, 5 or 10 parasites per cell. Data correspond to mean values of duplicates plus standard deviation. (**d,e**) Percentage of infected bone marrow derived MØ (**d**) and average of amastigotes per cell (**e**) at 24 and 72 hours post infection. WT or *LiRPIB* mutant stationary promastigotes were used at a ratio of 10 parasites per cell. At the appropriate time points, the cells were Giemsa stained and analysed microscopically. Data correspond to mean values of duplicates plus standard deviation. Two-tailed unpaired *t-*test was performed: statistical significance p < 0.05. The data displayed in **a–e** correspond to two independent experiments.

All promastigote mutants grew in a similar fashion to the WT (Fig. 4a). The expression of metacyclogenesis markers (Histone H4, SHERP, MET-1^26^) in stationary cultures was not statistically different from the WT (Fig. 4b). Moreover, all the clones successfully differentiated into axenic amastigotes and apparently enter bone marrow derived MØ normally, as observed at 4 hours post infection, either by FACS (Fig. 4c) or microscopic analysis. Indeed, no differences in the percentage of infected cells nor the numbers of parasites per infected cell were seen at 4 and 24 (Fig. 4d,e) hours post-infection between WT and mutant parasites. While the percentage of infected cells remained similar during experiments for all the mutants, cells infected with sKO presented a statistically significant decrease in the average number of amastigotes per cell at 72 hours post-infection (Fig. 4e). Indeed, cells infected with WT presented 2.19 ± 0.10 amastigotes per cell, while cells infected with sKO 1.66 ± 0.01. This reduction was due to a higher percentage of cells with one amastigote and a lower percentage of cells containing, at least, three amastigotes when compared with the WT (Supplementary Fig. S6b). Importantly, this phenotype is not observed in complemented sKO (sKO P15). Interestingly, no defects are seen in the growth of sKO 15 axenic amastigotes when compared to WT or sKO P15 (Supplementary Fig. S6c).

In conclusion, the *in vitro* results suggest that *Li*RPIB might be important for the replication of intracellular amastigotes.

### Impact of *Li*RPIB partial inactivation on the establishment of infection in mice

**Figure 5:**
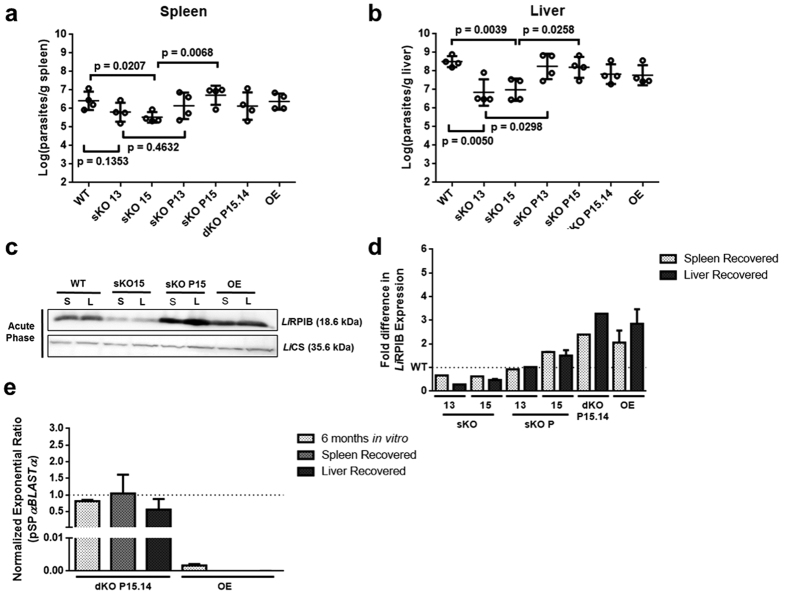
Infectivity of *LiRPIB* mutants in mice. **(a**,**b)** 1 × 10^8^ early stationary promastigotes were intra-peritoneally injected in BALB/c mice that were sacrificed 2 weeks post infection. The parasite burden in spleen (**a**) and liver (**b**) was assessed by the limiting dilution method. The values represent the mean of four animals ± standard deviation. Two-tailed unpaired *t* test was performed: statistical significance p < 0.05. (**c**,**d)** Western-blot analysis of RPIB levels in WT and *LiRPIB* mutants, which were recovered from the limiting dilution plates of the spleen (S) or liver (L). 1 × 10^7^ promastigotes were used for total extract preparation and *Li*CS (cysteine synthase) was detected as loading control. The fold difference in *of* RPIB expression in *LiRPIB* mutants in comparison to WT recovered from the spleen and liver at 2 weeks post-infection is shown in **d**. The results correspond to means plus standard deviation of two independent experiments. In **c**, a representative Western-blot is shown. **(e)** pSP*αBLASTα* quantification by qPCR using 10 ng of genomic DNA from *LiRPIB* OE and facilitated null mutant P15.14 that were left in culture for 6 months in the absence of blasticidin or recovered from spleen, or liver 2 months post-infection relative to the respective mutant maintained in culture under drug pressure. rRNA45 was used as a reference gene. The results correspond to means plus standard deviation of two independent experiments.

To evaluate the role of RPIB on the infectivity of the parasites, BALB/c mice were infected with WT or *LiRPIB* mutant promastigotes and the parasite burden in the spleen and liver determined at two (acute phase) and eight weeks (chronic phase) post infection. At two weeks post infection, differences in the splenic parasite loads were detected only for sKO mutant clone 15, while in the liver, animals infected with sKO mutants (clones 13 and 15) showed reduced parasite loads (Fig. 5a,b). Importantly, these differences were not seen in animals infected with sKO lines complemented with episomal *RPIB* (clones sKO P13 and sKO P15). Western-blot analysis of promastigotes differentiated back from the spleen and the liver in the acute phase demonstrates RPIB downregulation in sKO and overexpression in the complemented sKO. A representative Western-blot and the respective quantification are shown in Fig. 5c,d. In the chronic phase, there was overall a decrease of the parasite burden in the liver to levels close to the detection limit. This parasite clearance has been described in BALB/C model due to a partial self-resolution of the infection^27^. In the spleen, the parasite burdens were maintained similarly to the acute phase, and in the sKO mutants there was no parasite clearance or a more accentuated defect (data not shown). Overall both WT and mutant parasites persisted in both organs. Mutant parasites recovered from the spleen and the liver of chronically infected animals were analysed by qPCR for the presence of the episome carrying *RPIB*. While the OE had lost the episome over time, the same finding did not occur with the facilitated null mutant clone P15.14 (Fig. 5e), suggesting RPIB is also essential in amastigote form.

These data suggest RPIB is important in establishing *Leishmania* infection in the mouse, particularly in the liver.

### Assessment whether *Li*RPIB essentiality is dependent on the isomerase function

**Figure 6:**
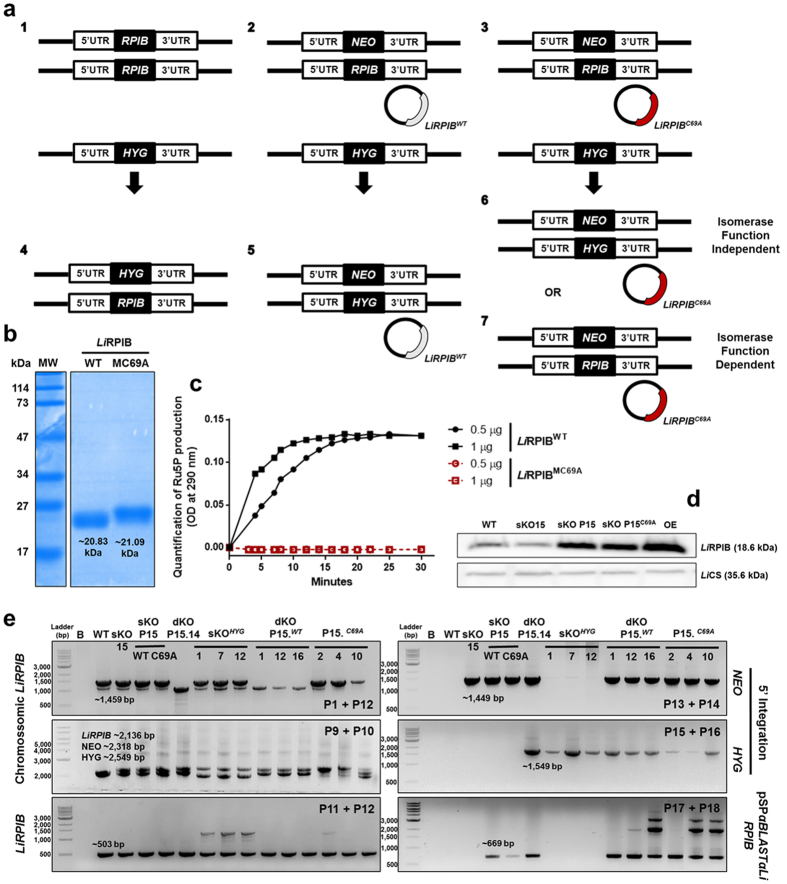
Assessing whether *Li*RPIB essentiality depends on its isomerase function. **(a)** Schematic overview of the strategy used to determine if *Li*RPIB essentiality depends on its isomerase function. WT (1), sKO 15 provided with pSP72*αBLASTαRPIBWT* (sKO P15^*WT*^, 2) or pSP72*αBLASTαRPIBC69A* (sKO P15^*C69A*^, 3) were transfected with a *HYG* containing construct. The generation of sKO*HYG* mutants from the transfection of the WT parasites (4), and the generation of facilitated null mutants from the transfection of sKO P15^*WT*^ parasites (5) were used as controls. The ability to remove the second *RPIB* allele or not when transfecting sKO P15^*C69A*^ parasites would determine whether the protein essentiality was independent (6) or dependent (7) on isomerase function, respectively. **(b)** Coomassie blue stained SDS-PAGE gel of 10 μg of recombinant *Li*RPIB^*WT*^ and *Li*RPIB^*C69A*^ post affinity chromatography purification. **(c)** Measurement of *Li*RPIB^*WT*^ and *Li*RPIB^*C69A*^ activity, catalysing the direct reaction (R5P−> Ru5P), using 50 mM of R5P and 0.5 or 1 μg of enzyme and the direct spectrophotometric method at 290 nm. The data are representative of 2 independent experiments. **(d)** Western-blot analysis of *Li*RPIB expression in WT, sKO 15, sKO P15^*WT*^, sKO P15^*C69A*^ and OE, before the transfection scheme represented in A. 1 × 10^7^ parasites were used for total extract preparation and *Li*CS (cysteine synthase) was used as loading control. **(e)** PCR analysis of *LiRPIB* mutants to assess *LiRPIB, NEO* and *HYG* presence in chromosome 28, as well as pSP*αBLASTαLiRPIB* presence. See Table S4 for primer sequences, the expected fragments and annealing sites are shown in Fig. 3s. B, blank.

We consider that assessing whether protein essentiality is due to the annotated metabolic function is particularly important in the context of drug target validation. *T. cruzi* RPIB isomerase activity is known to be abrogated by replacing cysteine 69 by an alanine without compromising the overall 3D conformation^16^. Therefore, we tested whether a similar mutation would abrogate *Li*RPIB isomerase activity and whether the removal of a second RPIB allele in a sKO complemented with a plasmid carrying the mutated RPIB (sKO P15^*C69A*^) would be successful. This experimental approach would reveal whether protein essentiality is independent (Fig. 6a6) or dependent (Fig. 6a7) on RPIB isomerase function, respectively.

For this purpose, a mutated recombinant *Li*RPIB^*C69A*^ protein was expressed in *E. coli* to confirm the inactivation of the isomerase function. The recombinant protein possessed the expected MW (~21.09 kDa, Fig. 6b) but failed to convert R5P into Ru5P (Fig. 6c) or Ru5P into R5P (data not shown). The sKO 15 was complemented with pSP72*αBLASTαRPIBC69A,* and the expression levels of RPIB in sKO P15^*WT*^ and sKO P15^*C69A*^ were found to be similar (Fig. 6d). Afterwards, WT (Fig. 6a1) and sKO 15 provided of pSP72*αBLASTαRPIBWT* (sKO P15^*WT*^, Fig. 6a2) or pSP72*αBLASTαRPIBC69A* (sKO P15^*C69A*^, Fig. 6a3) were transfected with a *HYG* construct (Fig. 6a). As controls, two series of mutants were successfully generated: sKO*HYG* mutants from WT transfection (Fig. 6a4) and facilitated null mutants from sKO P15^*WT*^ transfection (Fig. 6a5). Eleven out of 12 (Supplementary Table S2) facilitated null mutants were obtained from sKO P15^*WT*^ transfection, while no facilitated null mutants were obtained with sKO P15^*C69A*^. PCR analysis of representative mutants from each series is shown in Fig. 6e, following the same strategies depicted in Fig. 3a. The previously generated facilitated null mutant clone P15.14 was used as positive control.

These data indicate *Li*RPIB essentiality is due to its isomerase function.

### *TbRPIB* null mutant generation attempt by targeted gene replacement

**Figure 7:**
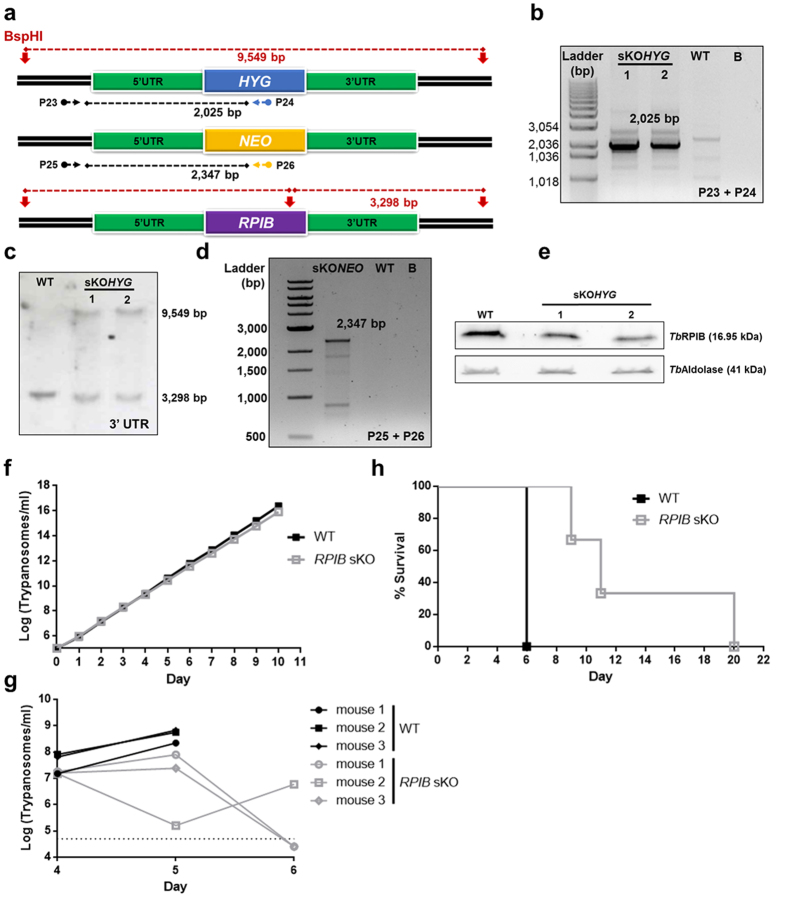
Generation *of TbRPIB* mutants and their *in vitro* and *in vivo* characterization. **(a)** *TbRPIB* locus (*RPIB* allele and targeted gene replacement cassettes, containing *NEO* and *HYG*) schematics. Horizontal arrows and numbers represent the primer pairs (sequences in Table S4) used to assess the genotype of the mutants: the black dashed line represents the expected PCR fragment. The colour of those arrows indicates the region of annealing: *HYG*, *NEO* or UTRs in blue, yellow or black, respectively. Southern-blot approach, upon digestion with *BspH*I (vertical red arrows) is also represented: dashed red lines represent the expected digestion fragments. **(b)** PCR analysis of *TbRPIB* sKO mutants to assess *HYG* 5′ integration. B, blank. **(c)** Southern-blot analysis of 10 μg of *TbRPIB* mutants (*versus* WT) genomic DNA, previously digested with *BspH*I, and probed using 3′ UTR. **(d)** PCR analysis of *TbRPIB* sKO mutant to assess *NEO* 5′ integration. B, blank. **(e)** Western-blot analysis of RPIB expression in bloodstream mutants (*versus* WT) using aldolase as loading control. **(f)** Growth curve of WT *versus* a representative sKO cell line. Black and grey squares represent WT and sKO growth, respectively. Cumulative cell numbers (product of cell number and total dilution) are plotted. Values represent averages from two independent experiments using one representative sKO clone and ± error bars indicate standard deviation. **(g)** Groups of mice (n = 3) were infected intraperitoneally with 1 × 10^4^ control WT (black) or a representative sKO clone (grey). Parasitemias of each animal are shown from 4^th^ to 6^th^ day post-infection. Values are means. 5 × 10^4^ trypanosomes/ml of blood is the detection limit, which is represented by the black dashed line. For *TbRPIB* sKO, the mean of parasitemia in two animals at day 6 was below the detection limit. Mice were culled when parasitemia reached 1 × 10^8^ cells/ml. **(h)** Kaplan–Meier survival analysis of mice infected with WT *versus* a representative sKO clone (black and grey line, respectively). Parasitemias and survival curve are representative of two independent experiments using two different sKO clones.

Taking into account RPIB essentiality in *L. infantum* and a previous report of its importance for *T. brucei* bloodstream forms infectivity^18^, we investigated whether essentiality would extend to this latter organism. To obtain *TbRPIB* null mutants, a target gene replacement strategy was also applied. One *TbRPIB* allele was replaced by *HYG* giving rise to sKO mutants at the first attempt. PCR and Southern blot, as shown in Fig. 7a, confirmed the correct insertion of the cassette (Fig. 7b,c). For the generation of *TbRPIB* dKO mutants, sKO parasites were transfected either with a *BLEO* construct or with a *NEO* construct, with no success. The *NEO* construct reliability was assessed by simultaneous transfection of WT and sKO mutant. Resistant parasites were only obtained for the WT but not for the sKO (Fig. 7d), enabling the generation of sKO but not null mutants. Therefore, we characterized the generated sKO cell lines, which showed a reduction in *Tb*RPIB protein levels of approximately 50% (semi-quantification by densitometry) in sKO cell lines in comparison with the WT, as assessed by Western-blot (Fig. 7e). No significant differences were found on the *in vitro* growth of *TbRPIB* sKO bloodstream forms compared with WT (Fig. 7f). *In vivo*, *TbRPIB* sKO showed a reduced infectivity, evaluated by the parasitaemia (Fig. 7g), consequently, the infected mice had an increased life span (Fig. 7h) when compared with WT parasites.

Our data strongly suggest that a functional copy of *TbRPIB* gene is essential for the parasite’s survival and that 50% decrease in RPIB levels is sufficient to compromise its infectivity.

## Discussion

The genomes of all the trypanosomatid parasites contain sequences that encode for a putative RPIB. Recombinant enzymes from *L. donovani*^17^, *T. brucei*^18^ and *T. cruzi*^9^ have been formally demonstrated to have *in vitro* isomerase activity, catalyzing the interconversion of R5P and Ru5P. In this paper we have demonstrated that the same applies to *L. infantum* and *L. major* homologues. *Leishmania* enzymes share over 90% identity with each other but only ~50% with the RPIB from trypanosomes. Nevertheless, the protein residues so far associated with isomerization, ring opening and phosphate charge stabilization are strictly conserved. The *K*_m_ values for both R5P and Ru5P were similar between *Leishmania* and trypanosomes, however, *k*_cat_ values are considerably higher for *Leishmania*, in both direct and reverse reactions (Supplementary Table 1 *versus*^9^,^17^,^18^). A decrease in *K*_m_ and an increase in *k*_cat_ were consistently observed for Ru5P in comparison to R5P (Supplementary Table S1), suggesting that the conversion of Ru5P into R5P is favoured, which might be explained by the important role of R5P as a building block for nucleic acid synthesis.

Trypanosomatids have unique organelles to respond to the specific needs of their life cycles. Amongst these are glycosomes, peroxisome-related organelles that comprise enzymes of important metabolic pathways such glycolysis, PPP, among others^28^. This compartmentalisation of metabolic pathways may prevent the accumulation of toxic intermediates^29^ or enable fast metabolic adaptation to environmental changes^28^. *Lm*RPIB possesses a peroxisome targeting sequence 2 (PTS-2), a glycosomal signal peptide sequence (–RVALGCDHA–30), that is conserved in both *L. infantum* and *L. donovani*. Our study suggests a dual localisation, despite most RPIB being detected in the cytosol of *L. infantum* promastigotes and amastigotes, it also localises to a certain extent in the glycosomes, similarly to what we have already described for *Tb*RPIB^18^. Moreover, other enzymes of the same pathway have been shown to display the same pattern, for example, those enzymes immediately upstream, or downstream, of RPIB, 6PGDH and TKL, respectively^11^,^12^,^30^,^31^,^32^. However, recent proteomic analysis of the *L. donovani* glycosomes failed to detect RPIB^33^. This same analysis detected HGPRT (PTS-1), aldolase (PTS-2), as well as some enzymes of the PPP, namely: putative G6PD, TKL, putative RPE (PTS-1), putative 6PGDH and putative TAL (non-identified signal peptide), or other related proteins such as putative ribokinase (PTS-2). We wonder whether this is due to the amount of RPIB in the glycosomes, or whether it is not present in all of them (Supplementary Fig. S3d).

To assess the importance of RPIB to *L. infantum* survival and infectivity, we attempted to generate null mutants. Successive failures to obtain mutants null to RPIB, coupled with constant aneuploidy generation were suggestive of gene essentiality^34^,^35^. We used a classical approach to demonstrate this^36^,^37^, by providing the sKO parasites with an episomal copy of *RPIB* and removing the second copy of the gene, we generated facilitated null mutants. Moreover, the plasmid was not lost even in the long-term absence of drug pressure both *in vitro* (Fig. 3f) and *in vivo* (Fig. 5e) further supporting the essentiality of RPIB for *L. infantum* survival in both promastigote and amastigote forms.

Due to the impossibility of generating mutants truly null to *RPIB*, we have performed phenotypic studies using the sKO lines. *In vitro,* sKO promastigotes grow, undergo metacyclogenesis, enter macrophages and differentiate normally. However, our data point to a defective replication of the intracellular amastigotes. Indeed, under these experimental conditions, amastigote replication can be evaluated as the number of parasites per cell significantly increases from 24 to 72 hours post-infection with the WT parasite. Importantly, MØ infected with sKO mutants showed a reduced number of amastigotes per cell 72h post-infection and this phenotype was rescued by complementation with episomal *RPIB*. It is noteworthy, that the remaining amount of protein probably suffices the parasite needs for minimal intracellular replication and therefore the overall effect is very modest. In fact, *in vivo*, an equally mild defect was observed, as expected considering the sKO lines present only a 50% downregulation in the protein levels (semi-quantification by densitometry). Moreover, there was no parasite clearance, not even at later stages of infection (data not shown), meaning that the parasites can persist. This type of phenotype is frequently described in mutants of *Leishmania* for enzymes involved in energy metabolism, e.g., glucose transporter knockout in *L. mexicana*^38^ and gluconeogenic enzyme fructose-1,6-bisphosphatase (FBPase) knockout in *L. major*^39^, as promastigotes are able to infect and differentiate, but the resulting amastigotes do not replicate failing to generate the normal lesions seen in the mouse model.

The higher expression of RPIB in amastigotes (Fig. 2a) and its importance for intracellular amastigote replication (Fig. 4e and Supplementary Fig. S6a,b) suggests a major role in this stage of the parasite’s life cycle.

Likewise, RPIB knockdown in *T. brucei* bloodstream forms, has been demonstrated *in vivo* to reduce infectivity, with an extension of survival for the murine host^18^. The defective phenotype observed *in vivo* for the *TbRPIB* sKO mutants (Fig. 7g,h) was less dramatic when compared to the one obtained with RNAi^18^. This might be explained by the more pronounced RPIB downregulation achieved with the RNAi system. Three independent attempts to generate dKO mutants have failed, suggesting that RPIB is essential for *T. brucei* survival. A *TbRPIB* conditional knockout would ultimately prove gene essentiality^40^. Nevertheless, our results support the hypothesis that RPIB is a potential therapeutic target in the fight against both *Leishmania* and *T. brucei* infections. However, critically analysing the *in vivo* data for both parasites, it appears that a high inhibition of the enzyme would be required to have a substantial impact on the infection, considering that for 50% downregulation the defects are modest, especially in the case of *Leishmania*.

Several enzymes have been reported to exhibit protein moonlighting in trypanosomatids^22^,^41^,^42^,^43^, some involved in carbohydrate metabolism^40^. In this sense, from a drug discovery perspective, it is critical to assess if the expected metabolic function is one that is detrimental to parasite survival and/or infectivity and therefore the one to be targeted by inhibitory molecules. We have unequivocally demonstrated that RPIB isomerase function is obligatory for the survival of *L. infantum* (Fig. 6).

We can only speculate why an enzyme involved in the non-oxidative branch of PPP, in which substrate interconversion takes place, should be essential. Amastigotes are known to have complex nutritional requirements, which may have precluded their establishment in early endosomal or non-hydrolytic vacuoles of MØ^43^, where the levels of amino acids and sugars are not sufficient for the parasite’s demands^44^,^45^. Incidentally, the variable nutritional composition of host cell phagolysosomes may be one of the reasons why the *Leishmania* promastigotes fail to differentiate, and the ensuing amastigotes, to replicate in neutrophils^2^, but are successful in MØs^43^. This may also explain why sKO mutants exhibit a more pronounced infectivity defect in the liver compared with the spleen.

Many *Leishmania* auxotrophs do not exhibit any loss of virulence and manage to persist in animal hosts, suggesting that the apparently hostile environment of the phagolysosome is somehow a permissive niche^43^. Importantly, amastigotes undergo a “stringent metabolic response”, characterized by a sharp decrease in glucose uptake that allows for more efficient energy metabolism, associated with a decrease of energy expenditure in anabolic processes and redirecting carbon to intracellular carbohydrate reserves^46^. It has been proposed that it facilitates long-term amastigote survival in the nutrient-limited environment of the phagolysosome, but simultaneously and paradoxically increasing dependency on carbohydrate uptake and metabolism^46^ Ribose can be imported^47^ and metabolized^48^ by *Leishmania* promastigotes and apart from its importance for nucleic acid synthesis it can also be an important source of energy for intracellular amastigotes^49^. The fact that RPIB sKO axenic amastigotes do not have any intrinsic defect in multiplication (Supplementary Fig. S6c), suggests glucose/ribose uptake from the phagolysosome might be a limiting factor. Interestingly in promastigotes, even upon D-ribose supplementation, the facilitated null mutants did not lose the plasmid carrying *RPIB* (Supplementary Fig. S5) suggesting that their ability to import ribose and/or convert it into R5P *via* ribokinase may not be sufficient to compensate RPIB ablation.

Moreover, organisms such as yeast exhibit an alternative NADP-independent pathway for R5P synthesis designated the riboneogenesis pathway^50^. Although not conclusively shown to be operational in trypanosomes, *Leishmania* lacks the sequence encoding for the putative key enzyme of the process^13^. The decrease in glucose and ribose importation along with the absence of an alternative pathway for RPIB generation would render the parasites more dependent on other enzymes that ultimately generate this metabolite.

Apart from the effects from the reduction in the R5P pool, absence of RPIB may lead to the potential accumulation of Ru5P. Indeed, if the upstream enzymes are regulated by the concentration of the end products, then the oxidative branch would become less operational leading to a decrease in NADPH production, rendering the parasites more susceptible to reactive oxygen species. Additionally, Ru5P accumulation could trigger another outcome, if we consider observations made in hepatocarcinoma cells, upon human RPIA knockdown^51^. In this study, the accumulated Ru5P was converted into xylulose-5P by RPE. This metabolite can activate PP2A activity^52^, which negatively regulates ERK signalling for cell proliferation. Whether this sort of regulation takes place in *Leishmania* has not been described, however, the genome does encode for putative RPE^19^,^20^, PP2A^53^ and several MAP kinases^54^.

In summary, RPIB, which lacks a human homologue, is essential for *L. infantum* survival and infectivity. We have indications that this may also be the case in *T. brucei*, thus taking a broader perspective RPIB may be considered a potential drug target in trypanosomatids. The fact that a simple *in vitro* activity assay could be used for the screening of inhibitory molecules coupled to the existence of structural data^16^, reinforce this protein as a good candidate. However, further studies concerning its druggability will need to be carried out.

## Materials and Methods

### Ethics statement

All experiments were approved by the IBMC. INEB Animal Ethics Committees and the Portuguese National Authorities for Animal Health guidelines, according to the statements on the directive 2010/63/EU of the European Parliament and of the Council.

### Chemicals and reagents

D-ribose-5-phosphate disodium salt hydrate, D-ribulose-5-phosphate disodium salt, D-ribose, EDTA, cysteinium chloride, tetracycline, carbazole, sulphuric acid, dNTPs, Tween-20, Tris-base, urea, thiourea, DTT, 2-mercaptoethanol, Triton X-100 and IPTG (isopropyl-β-D- thiogalactopyranoside) were purchased from Sigma. Oligonucleotide primers were obtained from STAB VIDA. Restriction endonucleases were from New England Biolabs. Polyclonal antibodies against *Li*RPIB and *Tb*RPIB were obtained in rabbits inoculated with purified recombinant His-tagged *Li*RPIB and *Tb*RPIB, respectively.

### Parasites

*L. infantum* (MHOM/MA/67/ITMAP-263) promastigote forms were cultured in complete RPMI 1640 or SDM media with or without supplementation with 100 μM of D-ribose at 26 °C. Axenic amastigote forms were cultured in complete MAA medium at 37 °C, 5% CO_2_, as described previously^26^. For *in vitro* and *in vivo* characterization, different cell lines were firstly recovered from the spleen of infected BALB/c to restore virulence, and posteriorly maintained in culture for no longer than 10 passages^26^. *T. brucei brucei* Lister 427 bloodstream forms were cultivated in HMI-9 medium, as previously described^55^. Depending on the analysis, protein extracts were prepared as follows: 1) 1 × 10^7^ late-stationary *L. infantum* promastigotes or *T. b. brucei* bloodstream forms were resuspended in T8 lysis buffer (Tris-base 0.6%, urea 42%, thiourea 15%, DTT 0.3%, Triton X-100 1%); or 2) 1 × 10^8^ promastigotes or axenic amastigotes were resuspended in 100 μL of PBS containing protease inhibitor (Roche) and following 6 freeze/thaw cycles, the parasite suspension supernatants were recovered and then quantified using Bio-Rad DC Protein Assay (Biorad). All the samples were stored at −80 °C.

### RPIB protein alignments

*Li*RPIB, *Lm*RPIB, *Tb*RPIB and *Tc*RPIB protein alignments were performed using the ClustalW program^56^. Aline program, *Version 011208*^57^ was used for editing protein sequence alignments.

### RPIB *in silico* localization prediction

*Li*RPIB subcellular localisation prediction was performed using WoLF PSORT^58^ and CELLO^59^.

### Cloning *RPIB* genes

Ribose 5-phosphate isomerase B from *L. infantum* (*LiRPIB*) and *L. major* (*LmRPIB*) was obtained by performing PCR on genomic DNA, extracted using DNAzol (Invitrogen) from *L. infantum* (MHOM/MA/67/ITMAP-263) and *L. major* strain Friedlin. Fragments of the open reading frames of *LiRPIB* (LinJ.28.2100; chromosome LinJ.28; 781,581–782,099) and *LmRPIB* (LmjF.28.1970; chromosome LmjF.28; 766,756–767,274)^19^,^20^ were PCR-amplified, using primers 1 + 2 and 3 + 4 (Supplementary Table S3), respectively. PCR conditions were as follows: initial denaturation (2 min at 94 °C), 35 cycles of denaturation (30 s at 94 °C), annealing (30 s at 45 °C,) elongation (2 min at 68 °C) and a final extension step (10 min at 68 °C). In the case of *LiRPIB*, another restriction strategy was required to clone the gene into a *Leishmania* overexpression vector – pSPα*BLAST*α (primers 7 + 8, Supplementary Table S3). PCR conditions were as follows: initial denaturation (2 min at 94 °C), 30 cycles of denaturation (15 s at 94 °C), annealing (30 s at 62 °C,) elongation (1 min at 72 °C) and a final extension step (10 min at 72 °C). In order to obtain *LiRPIB* with a point mutation with Cys69 (replaced by an alanine, C69A) – *LiRPIBC69A*, primers 5 + 6 and 7 + 6 (Supplementary Table S3) were used to amplify fragments of the ORF containing the restriction sites; *Xba*I/*Age*I or *Nhe*I/*Age*I and the desired point mutation, that were then cloned into pGEM-T in order to ultimately clone them in the pET28a(+) (Novagen) and pSPα*BLAST*α vectors, respectively. PCR conditions were as follows: initial denaturation (2 min at 94 °C), 35 cycles of denaturation (15 s at 94 °C), annealing (30 s at 62 °C,) elongation (30 s at 72 °C) and a final extension step (10 min at 72 °C). All the PCR products were obtained using a Taq DNA polymerase with proofreading activity (Roche), isolated, cloned into a pGEM-T Easy vector (Promega) and sequenced.

### Expression and purification of recombinant *Li*RPIB (WT and C69A) and *Lm*RPIB

The *LiRPIB*^*WT*^, *LiRPIB*^*C69A*^ and *LmRPIB* genes were excised from the pGEM-T Easy vector (using *Nde*I/*EcoR*I, *Nhe*I/*Sac*I and *Nde*I/*EcoR*I, respectively), gel purified and subcloned into pET28a(+) expression vector. The resulting constructs presented a 6-histidine tag at the N-terminal and were transformed into *E. coli* BL21DE3 cells. Proteins were purified by affinity chromatography as previously described^18^. Concentration was determined by measuring absorbance at 280 nm using the theoretical molar extinction coefficients of 12950, 12950 and 7450 M^−1^.cm^−1^ for *Li*RPIB^WT^, *Li*RPIB^C69A^ and *Lm*RPIB, respectively, making use of NanoDrop ND-1000 Spectrophotometer (NanoDrop Technologies). Ten μg of purified recombinant proteins were resolved in SDS/PAGE stained with Coomassie Brilliant Blue G-250 (Biorad).

### Western-blot analysis

Five hundred ng of *Li*RPIB^WT^ recombinant protein, 20 μg of total soluble extracts from both promastigote and amastigote forms, or 1 × 10^7^ parasites were resolved in SDS-PAGE and transferred onto a nitrocellulose membrane (TransBlot Turbo, Bio-Rad), which was blocked, probed, washed and developed as previously described^18^. The following primary antibodies were used: mouse anti-α-tubulin (clone DM1A, Neomarkers, 1:1000), rabbit anti-*Li*RPIB (1:500 or 1:1000), rabbit anti-*Tb*RPIB (1:1000), rabbit anti-*Li*CS (cysteine synthase, 1:2000), rabbit anti-*Ld*HGPRT (hypoxanthine guanine phosphoribosyl transferase, 1:2000), rabbit anti-*Tb*Enolase (1:5000), abbit anti-*Tb*Aldolase (1:5000), rabbit anti-*Li*mTXNPx (mitochondrial peroxiredoxin, 1:1000) and rabbit anti-*Li*AS-A (asparagine synthetase A, 1:1000). *Horseradish* peroxidase-conjugated goat anti-rabbit or goat anti-mouse IgG (Amersham) (1:5000 for 1 h, at RT) were used as the secondary antibody. ImageJ software (version 1.47) was used for protein semi-quantification.

### Enzymatic Assay

To determine the *K*_m_ for R5P and for Ru5P, a direct spectrophotometric method at 290 nm or a modification of Dische’s Cysteine-Carbazole method were used, respectively^9^. The experimental set up was performed as previously^18^.

### Generation of *LiRPIB* overexpressed (OE) and facilitated null mutants

A targeted gene replacement strategy was used for *L. infantum RPIB* gene knockout. All the primer sequences for gene replacement cassettes and confirmation of the mutants’ genotypes are given in Supplementary Table S4. Briefly, *RPIB* flanking regions were amplified from *L. infantum* genomic DNA and were linked to neomycin phosphotransferase (*NEO*) or hygromycin phosphotransferase (*HYG*) genes using a fusion PCR approach. The 5′ and 3′ UTR were amplified using primers 1 + 2 and 3 + 4, respectively. *NEO* and *HY*G were amplified from pSP72*αNEOα* and pGL345*HYG* templates, using primers 5 + 6 and 7 + 8 respectively, which possess around 30 nucleotides of the 5′ UTR in the sense primer and the first 30 nucleotides of the 3′ UTR in the antisense primer. 5′ UTR_*NEO_*3′ UTR and 5′ UTR_*HYG*_3′ UTR constructs were obtained using primers 9 + 10. To obtain an episomal copy of *RPIB*, *LiRPIB*^*WT*^ or *LiRPIB*^*C69A*^, genes were excised from the pGEM-T Easy vector (using *Xba*I/*Nde*I), gel purified and subcloned into the vector pSP72*αBLASTα*. Approximately 10 μg of either linear fragments obtained by fusion PCR or plasmid were purified, concentrated and transfected into 5 × 10^7^ mid-log promastigotes, using an Amaxa Nucleofector II device with human T-cell nucleofector kit (Lonza). The day after transfection, drug selection was carried out at 20 μg/mL of G418 (Invitrogen), 50 μg/mL of hygromycin B (InVivoGen) and/or 30 μg/mL blasticidin (InVivoGen). Parasite cloning, except for transfections with episomes was performed by diluting the parasite suspension to a concentration of 0.5 cells per well in 96-well plates using SDM culture medium. Mutants overexpressing *LiRPIB* (OE) were obtained by transfecting WT parasites with pSP72*αBLASTα LiRPIB*^*WT*^. The facilitated null mutants were generated by complementing sKO mutants with pSP72*αBLASTαLiRPIB*^*WT*^, followed transfection with 5′ UTR_*HYG*_3′ UTR.

### Generation of *TbRPIB* mutants

Three different DNA cassettes were generated. The DNA fragments consisted ORFs of the resistance genes (hygromycin, bleomycin and neomycin), flanked by the 5′ and 3′ UTRs of the *TbRPIB* gene. For the generation of sKO mutants, the plasmid used was *pGL345-HYG* modified using the 5′ (924 bp, primers 19 + 20, Supplementary Table S4) and 3′ (997 bp, primers 21 + 22, Supplementary Table S4) *RPIB* flanks obtained through PCR from *T. brucei* genomic DNA template. The primers contained *Hin*dIII/*Sal*I and *Sma*I/*BgI*II restriction sites were used for cloning into the appropriate pre-digested pGL345*HYG*. To remove the second allele, pGL345*BLEO* or pGL345*NEO* plasmids were constructed by replacing the *HYG* with *BLEO* or *NEO*, respectively, in the plasmid used to obtain the sKOs, employing *Spe*I/*Bam*HI restriction enzymes. Thereafter the final plasmids were digested with HindIII and BglII to obtain the final DNA fragment for transfection. Transfection was performed as described for *LiRPIB* mutants, using 10 μg of DNA. Selection was undertaken using 7.5 μg/ml, 0.2 μg/ml and 5 μg/ml of hygromycin, bleomycin or neomycin, respectively.

### PCR and Southern-blot analysis of *RPIB* mutants

*LiRPIB* mutants were analysed by PCR (NZYTech or Invitrogen Taq Polymerase) for the presence of the following events: *LiRPIB* and selectable markers, *LiRPIB*, *LiRPIB* 5′ integration, *NEO* 5′ integration, *HYG* 5′ integration and pSP72*αBLASTαLiRPIB*, using primers pairs 9 + 10, 11 + 12, 1 + 12 and 9 + 12, 13 + 14, 15 + 16 and 17 + 18, respectively (Supplementary Table S4). *TbRPIB* mutants were analysed by PCR for the following events: *HYG* and *NEO* 5′ integration, using primers pairs 23 + 24, 25 + 26, respectively (Supplementary Table S4). For Southern blot (SB) analysis, total genomic DNA was extracted. Ten μg of genomic DNA were digested O/N with a 5 fold excess of *EcoR*I and *Nae*I (*Li*RPIB mutants) or *BspH*I (*TbRPIB* mutants) at 37 °C and samples were run O/N in a 0.8% agarose gel. The transfer into a nylan membrane, nucleic acid fixation, hybridisation and revelation were performed as previously described^21^. In the case of *LiRPIB* mutants, the blots were probed sequentially with 3′ UTR, *LiRPIB* (Fig. 3c), *NEO* and *HYG*, which were PCR amplified, using primers 3 + 4, 11 + 12, 5 + 6 and 7 + 8 (Supplementary Table S4), respectively. In the case of *TbRPIB* mutants, the blots were probed with 3′ UTR (Fig. 7d), amplified using primers 21 + 22 (Supplementary Table S4).

### Real-Time quantitative PCR (qPCR) analysis of *LiRPIB* mutants

qPCR analysis was used to assess the expression of metacyclogenesis markers in *LiRPIB* promastigote mutants, as well as pSP72*αBLASTαLiRPIB*^*WT*^ copy number. Concerning the expression of the markers for metacyclogenesis (MET1, Histone H4, SHERP (small hydrophilic endoplasmic reticulum associated protein)), total RNA extraction, reverse transcription and qPCR were performed as shown previously^26^. To assess pSP72*αBLASTαLiRPIB*^*WT*^ copy number, 10 ng of genomic DNA, primers 17 + 18 (Supplementary Table S4), and purified plasmid (positive control) were used. In both cases, rRNA45 was used as a reference gene and qPCR reactions were run in duplicate for each sample on a Bio-Rad My Cycler iQ5 (BioRad).

### *In vitro* growth of *RPIB* mutants

Growth curves of *LiRPIB* mutants and WT were seeded at 1 × 10^6^ parasites/ml in complete RPMI or complete MAA for promastigote and axenic amastigote forms, respectively. Before launching growth curves, the parasites were maintained in log phase for 2–3 passages in the absence of selection. For *TbRPIB* mutants and WT *in vitro* growth curves, cell lines were seeded at 1 × 10^5^ parasites/ml of complete HMI-9 medium, after 48h in the absence of selective drugs. Every 24 h, until day 7 or day 10 in the case of *L. infantum* or *T brucei*, respectively, cell growth was monitored microscopically.

### *In vitro* bone marrow derived macrophages (BMMø) infection by *LiRPIB* mutants

Cell suspension of bone marrow was obtained by flushing the femurs of susceptible BALB/c mice and culturing in 96-well plates (1 × 10^5^ cells per well) or in 24-well plates containing cover glasses (3.5 × 10^5^ cells per well) in LCCM supplemented DMEM, as previously^26^. For FACS analysis, CFSE labelled promastigotes^26^ were incubated with the BMMø at ratios of 2:1, 5:1 and 10:1 during 4 hours and then cells were washed to remove non-internalized parasites. The infection rates were determined at 4, 24 and 48 hours post-infection using the BD FACS Canto II cytometer and analysed by FlowJo software. For microscopic analysis, at day 7 of culture, promastigotes were incubated with the BMMø at a 10:1 ratio and again after 4 hours, infection was stopped. Infection ratios and number of parasites per cell were assessed at 4, 24 and 72 hours post infection. At these time points, cells were fixed with 3% *p*-formaldehyde (PFA), Giemsa stained and cover glasses were mounted on slides, using Vectashield (Vector Labs). Microscopic analysis was performed using a Nikon eclipse 80i (Nikon).

### *In vivo* infectivity of *RPIB* mutants

To characterize *LiRPIB* mutants, five to six weeks old female BALB/c mice were obtained from Charles River. For mouse infections, promastigotes from 4 days old stationary cultures were collected, washed, resuspended in PBS to a final amount of 1 × 10^8^ parasites/animal, and injected intraperitoneally. Mice were sacrificed at 2 or 8 weeks post-infection. The parasite burden in the spleen and liver was determined by limiting dilution as previously described^60^. To characterize *TbRPIB* mutants, after 48h in the absence of selective drugs, 1 × 10^4^ WT and sKO parasites were inoculated intraperitoneally into 6–8 week old BALB/c mice. Parasitaemia was measured on the fourth, fifth and sixth day post-infection through tail blood extraction.

### Digitonin Fractionation

This procedure has been performed as previously described^21^. All fractions were analysed by Western-blot.

### Immunofluorescence

*L. infantum* mid-log or late stationary promastigotes and axenic amastigotes were fixed, permeabilised and stained as previously described^61^. Parasites were spread on 8 well-IF slides (Polysciences) or 18 well-IF slides (Ibidi) for wide field and confocal microscopy, respectively. The following primary antibodies were used: rabbit anti-*Li*RPIB (1:500), sheep anti-*Li*TDR1 (1:500) and rabbit anti-*Li*HGPRT (1:500). The following secondary antibodies were used: goat anti-rabbit Alexa Fluor 488 or Alexa Fluor 568 and donkey anti-sheep Alexa Fluor 488 (Molecular probes, Life Technologies). In the case of RPIB and HGPRT colocalisation studies, parasites were sequentially incubated with rabbit anti-*Li*RPIB O/N at 4 °C, unconjugated sheep anti-rabbit for 60 min at RT, donkey anti-sheep Alexa Fluor 488 for 60 min at RT, rabbit anti-*Li*HGPRT for 2 hours at RT and goat anti-rabbit Alexa Fluor 568 for 60 min at RT (sequential stainings lacking only anti-*Li*RPIB or anti-*Li*HGPRT as well as single staining of each of the proteins were performed simultaneously as controls). Images were captured using wide field fluorescence microscope AxioImager Z1 (Carl Zeiss) and confocal Leica TCS SP5II microscope (Leica) for antibody validation and colocalisation studies, respectively. Images were analysed using ImageJ (version 1.47) or Fiji (version 1.45) software’s.

### Statistical Analysis

For statistical analysis, two-tailed unpaired *t*-test was used. Statistical analysis was performed using GraphPad Prism Software (version 5.0) and significance was found when p < 0.05.

## Additional Information

**How to cite this article**: Faria, J. *et al.* Disclosing the essentiality of ribose-5-phosphate isomerase B in Trypanosomatids. *Sci. Rep.* **6**, 26937; doi: 10.1038/srep26937 (2016).

## Supplementary Material

Supplementary Information
