# Supplementary material for: Disclosing the essentiality of ribose-5-phosphate isomerase B in Trypanosomatids
Source: Sci Rep. 2016 May 27;6:26937. doi: 10.1038/srep26937 (PMC4882579; doi:10.1038/srep26937)
Supplement: Supplementary Information [file srep26937-s1.pdf]

Supplemental Material

"Disclosing the essentiality of ribose-5-phosphate isomerase B in Trypanosomatids"

Joana Faria[1,2], Inês Loureiro[1,2], Nuno Santarém[1,2], Pedro Cecílio[1,2], Sandra Macedo-Ribeiro[2,3], Joana Tavares[1,2†*] & Anabela Cordeiro-da-Silva[1,2,4†*]

[1]Parasite Disease Group, Instituto de Biologia Molecular e Celular da Universidade do Porto, Portugal

[2]Instituto de Investigação e Inovação em Saúde, Universidade do Porto, Porto, Portugal

[3]Protein Crystallography Group, Instituto de Biologia Molecular e Celular da Universidade do Porto, Portugal

[4]Departamento de Ciências Biológicas, Faculdade de Farmácia, Universidade do Porto, Portugal

[*]Corresponding authors: jtavares@ibmc.up.pt or cordeiro@ibmc.up.pt

[†]These authors contributed equally to this work

Supplementary Figure S1

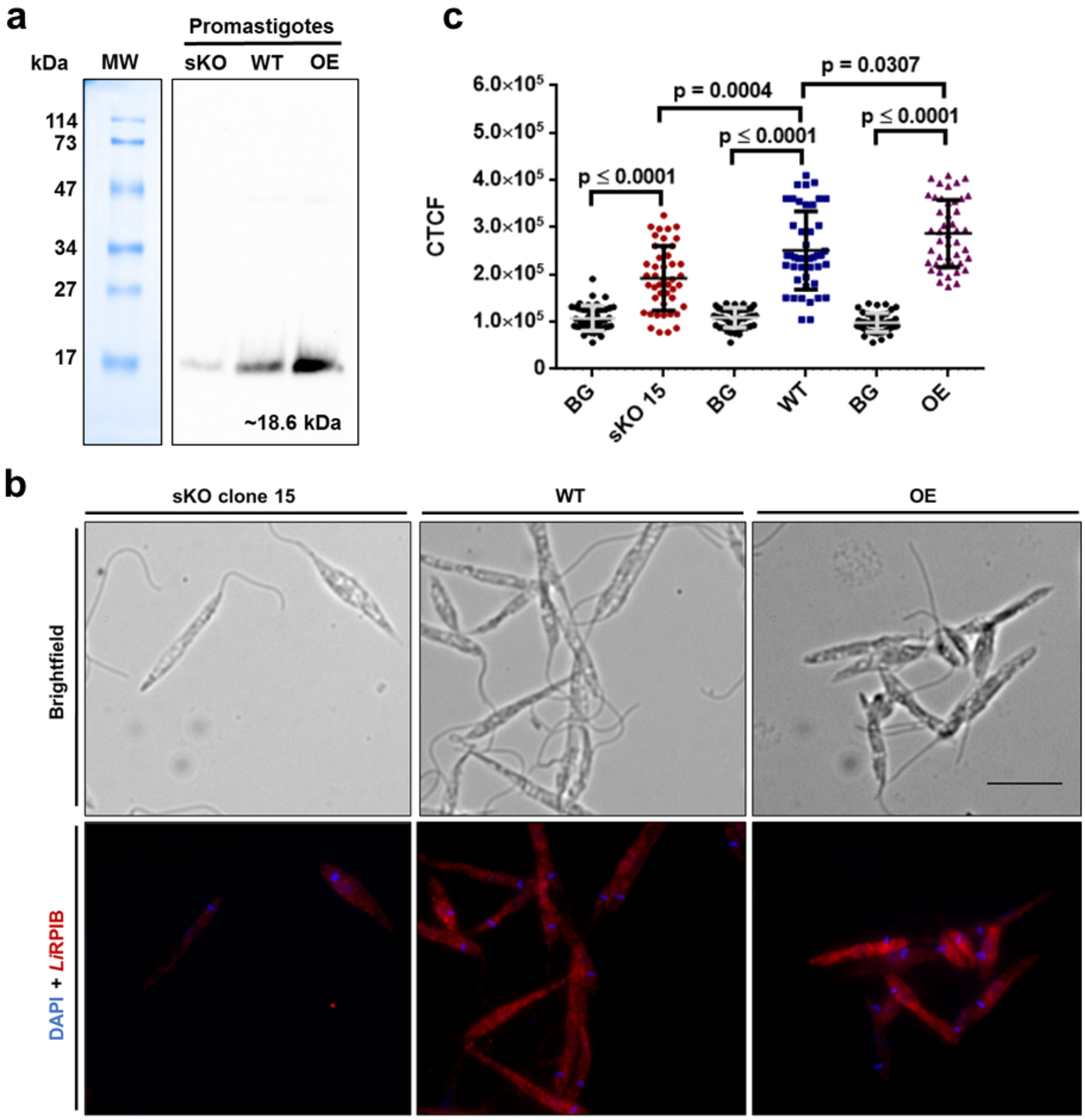

Figure S1. Rabbit polyclonal anti-*Li*RPIB antibodies validation. (a) Western-blot analysis of WT, *LiRPIB* sKO 15 and OE promastigotes extracts using rabbit polyclonal anti-*Li*RPIB (1:500). **(b)** Representative immunofluorescence images of different genotypes (WT, sKO 15 and OE) of mid-log *L. infantum* promastigotes, using rabbit polyclonal anti-*Li*RPIB antibody (1:500). Upper and lower panels present brightfield and *Li*RPIB (red) + DAPI (blue) stained images, respectively. Images were acquired with a 63x objective, using a Zeiss AxioImager Z1. The scale bar corresponds to 5 μm. **(c)** Fluorescence intensity quantification in WT, sKO

15 and OE parasites when stained with anti-*Li*RPIB antibody (1:500). The values are expressed in CTCF (corrected total cell fluorescence), and background (BG) values are displayed as well. The quantification was performed on images acquired with 63x objective, using a Zeiss AxioImager Z1 and the same exposure time for all genotypes (*Li*RPIB 300 ms; DAPI 100 ms). Twenty different fields for each genotype were analysed in duplicate, and the fluorescence of an average of 50-100 parasites was quantified using ImageJ (v 1.47) software. Two-tailed unpaired *t* test was performed: statistical significance $p < 0.05$. The results (a-c) are representative of 2 independent experiments.

Supplementary Figure S2

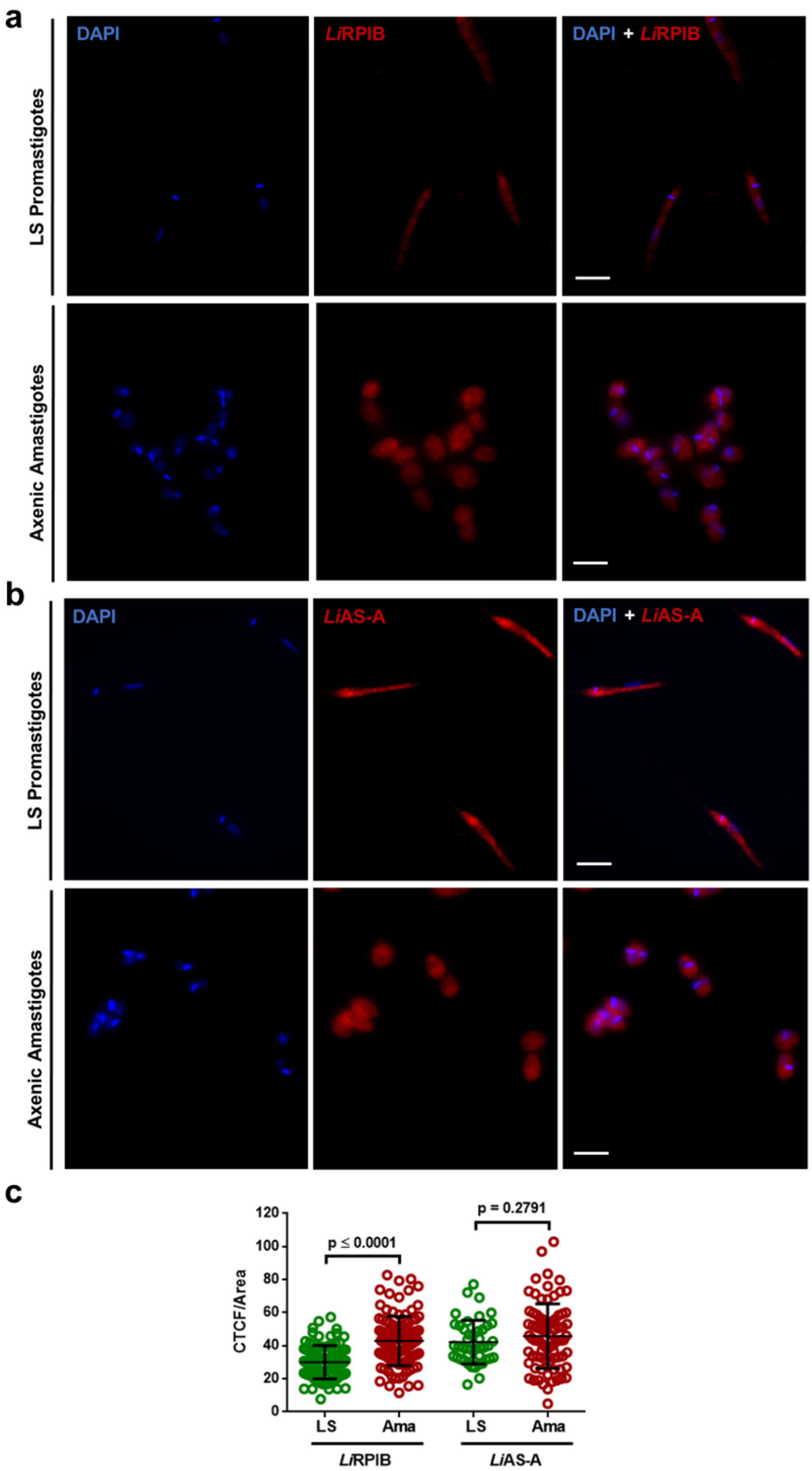

Figure S2. Quantification *Li*RPIB expression in promastigotes and amastigotes by immunofluorescence analysis. (a-b) Immunofluorescence analysis showing RPIB (red, in a) and AS-A (red, in b) localization in *L. infantum* promastigotes (upper panel) and axenic amastigotes (lower panel). Nucleus and kinetoplast DNA were stained with DAPI (blue). RPIB and AS-A were stained using rabbit polyclonal anti-*Li*RPIB (1:500) or anti-*Li*AS-A (1:1000) antibodies, respectively. Images were acquired with a 100x objective, using a Zeiss AxioImager Z1. The scale bar corresponds to 5 μm. **(c)** Fluorescence intensity quantification in late stationary promastigotes (LS) and axenic amastigote (Ama) forms when stained with anti-*Li*RPIB or anti-*Li*AS-A antibodies. The values are expressed in CTCF/area, which represents the corrected total cell fluorescence normalized for the parasite area. The quantification was performed on images acquired with 100x objective, using a Zeiss AxioImager Z1 and the same exposure time for both parasite forms (600 ms; DAPI 100 ms). Twenty different fields for each parasite form were analysed in duplicate, and the fluorescence of an average of 50-100 parasites was quantified using ImageJ (v 1.47) software. Two-tailed unpaired *t* test was performed: statistical significance $p < 0.05$. The results (a-c) are representative of 2 independent experiments.

Supplementary Figure S3

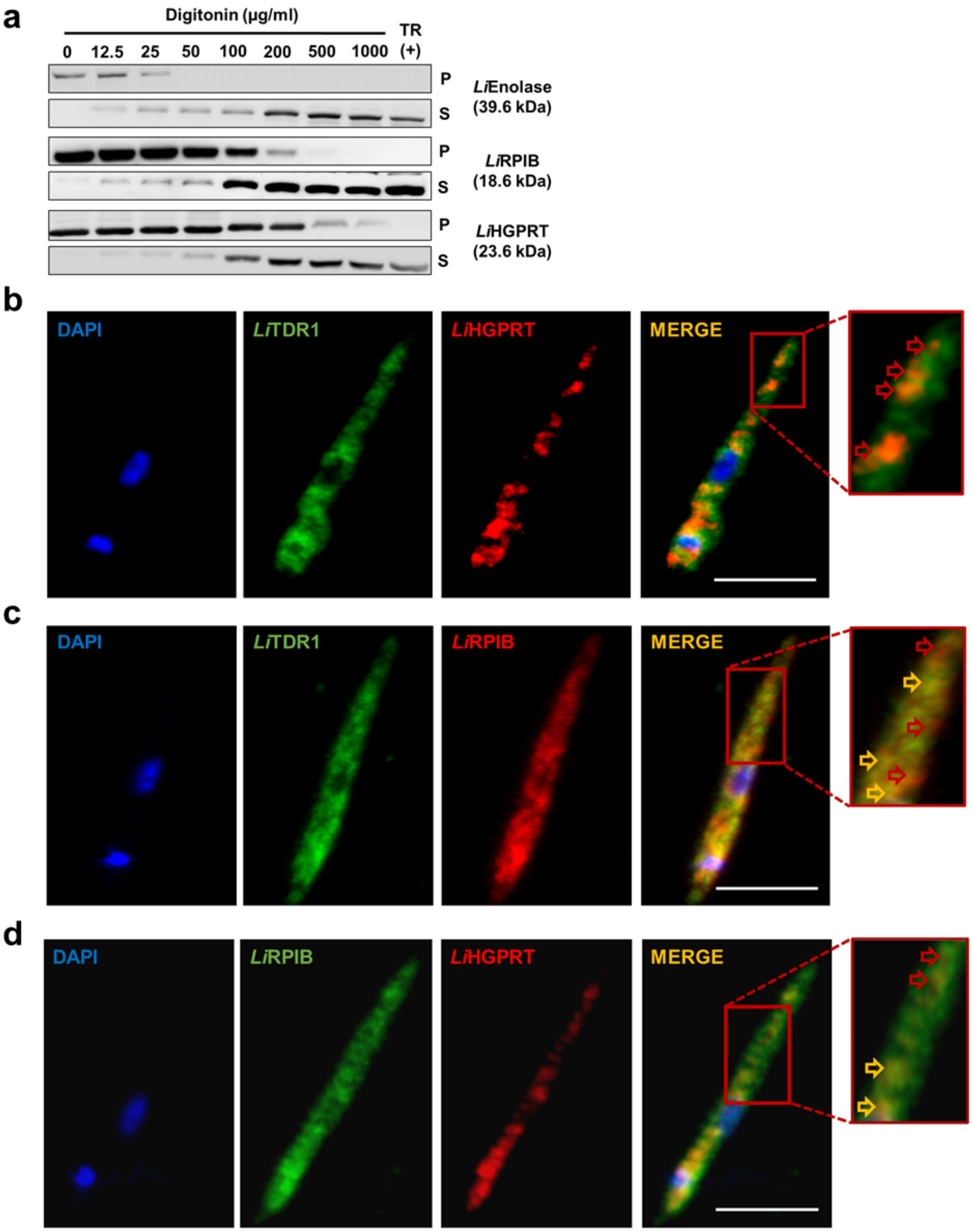

Figure S3. RPIB localization in *L. infantum* promastigotes. (a) Digitonin fractionation of mid-log promastigotes. Pellet (P) and supernatant (S) fractions obtained using increasing concentrations of digitonin or positive control with 1% of Triton X-100 (TR – Total Release) were subjected to Western-blot analysis and probed with antibodies against *Li*RPIB,

*Li*Enolase (cytosolic marker) and hypoxanthine guanine phosphoribosyltransferase *Li*HGPRT (glycosomal marker). **(b-d)** Immunofluorescence analysis showing RPIB (red in c; green in d) localizationin *L. infantum* promastigotes. Nucleus and kinetoplast DNA, cytosol and glycosomes were stained with DAPI (blue), anti-*Li*TDR1 (thiol-dependent reductase 1, green) and anti-*Li*HGPRT (hypoxanthine guanine phosphoribosyltransferase, red), respectively. On panels c and d, yellow arrows in the zoomed areas point to colocalisation sites, and the red arrows point to sites of exclusive *Li*RPIB (c) or *Li*HGPRT (a and d) staining. Images are maximal Z-projections of 30 to 35 contiguous stacks separated by 0.1 µm and were acquired with a 63x objective, using a LEICA SP5II confocal microscope. The scale bar corresponds to 5 µm. Data displayed a-d are representative of three independent experiments.

Supplementary Figure S4

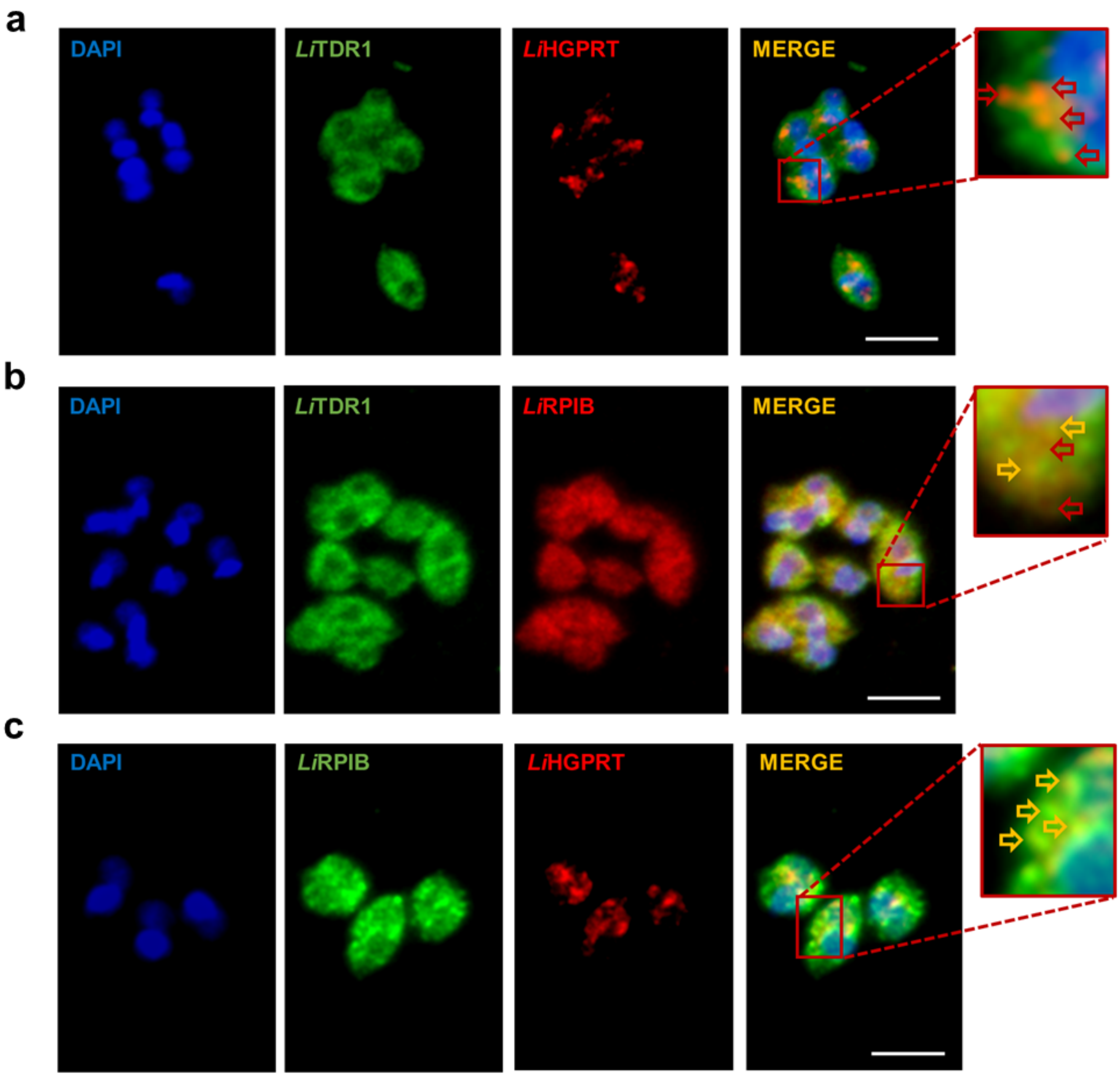

Figure S4. RPIB localization in *L. infantum* amastigotes. (a-c) Immunofluorescence analysis showing RPIB (red in b; green in c) localization. Nucleus and kinetoplast DNA, cytosol and glycosomes were stained with DAPI (blue), anti-*Li*TDR1 (thiol-dependent reductase 1, green) and anti-*Li*HGPRT (hypoxanthine guanine phosphoribosyltransferase, red), respectively. In panels b and c, the yellow arrows in the zoomed areas point to colocalisation sites, the red arrows point to sites of exclusive *Li*RPIB (b) or *Li*HGPRT (a) staining. Images are maximal Z-projections of 30 to 35 contiguous stacks separated by 0.1 µm and were acquired with a 63x objective, using a LEICA SP5II confocal microscope. The

scale bar corresponds to 5 μm. Data displayed a-c are representative of three independent experiments.

Supplementary Figure S5

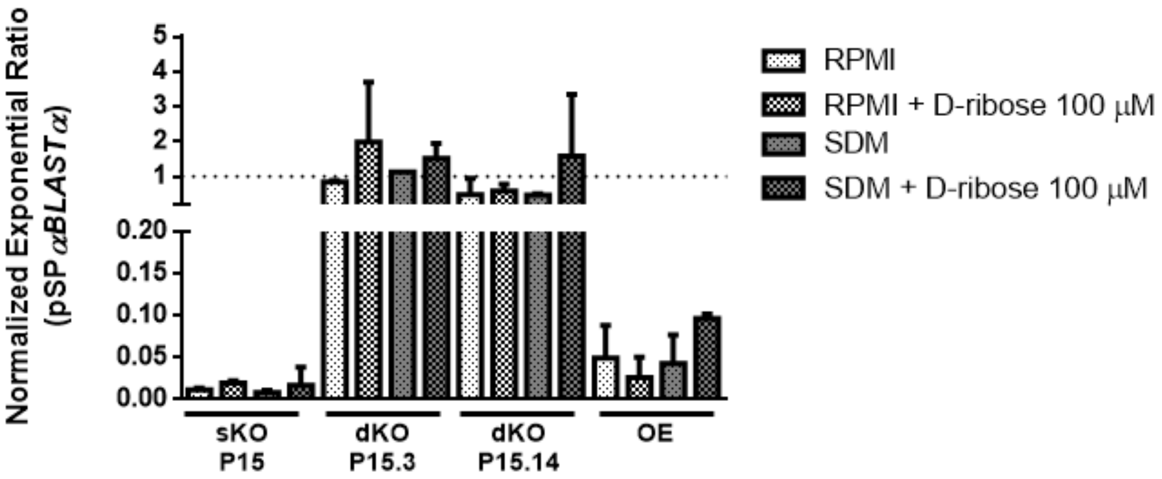

Figure S5. pSP*αBLASTαRPIB* retention or loss upon ribose supplementation. pSP*αBLASTαRPIB* quantification by qPCR using 10 ng of genomic DNA from *LiRPIB* sKO P15, facilitated null mutants P15.3 and P15.14 and OE. The DNA was extracted from parasites maintained in logarithmic phase for eight weeks in complete RPMI or SDM media with or without D-ribose supplementation, with or without blasticidin. For each mutant, the calibration was performed against the correspondent mutant maintained in culture in the presence of blasticidin. rRNA45 was used as the reference gene. The results correspond to means plus standard deviation of two independent experiments.

Supplementary Figure S6

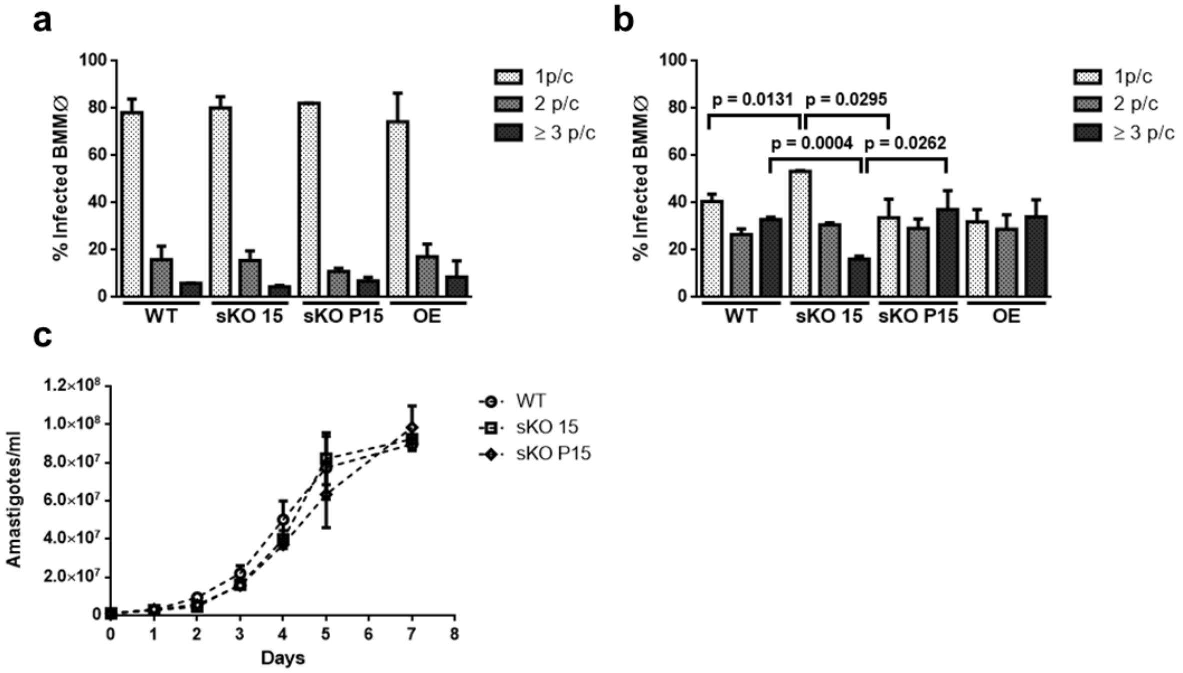

Figure S6. *In vitro* characterization of *LiRPIB* mutants: amastigote replication. (a and b) Percentage of infected bone marrow derived MØs that contain 1, 2 or ≥ 3 parasites, at 24 (a) and 72 (b) hours post infection. WT or *LiRPIB* mutant stationary promastigotes were used at a ratio of 10 parasites per cell. At the appropriate time points, the cells were Giemsa stained and analysed microscopically. Data correspond to mean values of duplicates plus standard deviation. Two-tailed unpaired *t*-test was performed: statistical significance $p < 0.05$. The data correspond to two independent experiments. **(c)** *L. infantum* axenic amastigotes growth curves of *LiRPIB* mutants (*versus* WT), cultured in complete MAA. Data correspond to mean values of duplicates ± standard deviation and are representative of two independent experiments.

Supplementary Table S1. Kinetic parameters of *Li*RPIB and *Lm*RPIB for R5P and Ru5P in the direct and reverse reaction, respectively

Species	R5P -> Ru5P				Ru5P -> R5P			
	K_m (mM)	v_{max} (mM.s^{-1})	k_{cat} (s^{-1})	K_{sp}* (M^{-1}.s^{-1})	K_m (mM)	v_{max} (mM.s^{-1})	k_{cat} (s^{-1})	K_{sp}* (M^{-1}.s^{-1})
***Li*RPIB**	4.34 ± 0.62	$3.90 \times 10^{-3} \pm 1.44 \times 10^{-4}$	43.49 ± 1.61	1.00×10^{4}	2.82 ± 0.30	$1.55 \times 10^{-2} \pm 5.20 \times 10^{-4}$	115.30 ± 3.86	4.09×10^{4}
***Lm*RPIB**	5.59 ± 0.75	$3.86 \times 10^{-3} \pm 1.48 \times 10^{-4}$	43.11 ± 1.65	7.71×10^{3}	3.01 ± 0.54	$1.73 \times 10^{-2} \pm 9.86 \times 10^{-4}$	128.50 ± 7.33	4.27×10^{4}

* K_{sp} Specificity Constant (k_{cat}/ K_m)
The values are means ± standard deviations obtained from 3 independent experiments.

Supplementary Table S2. Summary of the different cell lines obtained in different attempts to generate *LiRPIB* null mutants

Clone	Construct	dKO clones	Aneuploid clones
sKO*HYG* clone 4	*NEO*	0/12	6/12
sKO*HYG* clone 4	*NEO*	0/10	3/10
sKO*NEO* clone 13	*HYG*	0/7	3/7
sKO*NEO* clone 15	*HYG*	0/7	3/7
sKO*NEO* clone 15 + pSP72*αBLASTαLiRPIBWT*	*HYG*	11/12	1/12
sKO*NEO* clone 15 + pSP72*αBLASTαLiRPIBC69A*	*HYG*	0/11	7/11

Supplementary Table S3. Oligonucleotides sequences used to obtain *Li*RPIBWT (P1 + P2), *Lm*RPIB (P3 + P4), *Li*RPIBC69A (P5 + P6) recombinant proteins, pSPα*BLAST*α*LiRPIB* (P7 + P8) and pSPα*BLAST*α*LiRPIBC69A* (P7 + P6)

Primer	Sequence
**1**	5’ CAATTTCCATATGCCGAAGCGTGTTGC 3’
**2**	5’ CCCAAGCGAATTCTCTACTTTCCTTCC 3’
**3**	5’ CAATTTCCATATGTCGAAGCGTGTTGCTC 3’
**4**	5’ CGGATGCGAATTCTCACTTTCCTTCTTGG 3’
**5**	5' GGCTAGCATGCCGAAGCGTGTTGCTCTG 3'
**6**	5' TGCCGATACCGGTGCCTGCGACAAGGATAC 3'
**7**	5' GTCTAGAATGCCGAAGCGTGTTGCTCTG 3'
**8**	5' GCGCATATGTCACTTTCCTTCCTCCTTAAGACC 3'

Table S4. Oligonucleotide sequences used to obtain gene replacement cassettes (*L. infantum* P1-P10; *T. brucei* P19-P22) and to confirm the genotype of the *RPIB* mutants (*L. infantum* P1, P9-18; *T. brucei* P23-P26)

Primer	Sequence
**1**	5' TTCGAGAGCGGGATGGAGAG 3'
**2**	5' AGGGTGGATGGCTGGATGAG 3'
**3**	5' CACAAGGCGATGGGTACAAG 3'
**4**	5' GCACACGAGGTGCAGCAATG 3'
**5**	5' AGCGCTCTCTCTCCATCCCGCTCTCGAAATGATTGAACAAGATGGATTGC 3'
**6**	5' GATGAGGCCAACGGCCTTGTACCCATCGCCTTGTGTCAGAAGAACTCGTC 3'
**7**	5' CACCACCAAGCGCTCTCTCTCCATCCCGCTCTCGAAATGAAAAAGCCTGAACTCAC 3'
**8**	5’ TATGATGAGGCCAACGGCCTTGTACCCATCGCCTTGTGCTATTCCTTTGCCCTCGGAC 3’
**9**	5’ AGGGTGGATGGCTGGATGAG 3’
**10**	5' GCACACGAGGTGCAGCAATG 3'
**11**	5' ATGCCGAAGCGTGTTGCTCTG 3'
**12**	5' TTAAGACCTCCACAACCGCTGAAG 3'
**13**	5' CCTGCTTCGTAGCCTGTGCAAGTC 3'
**14**	5' GTGGTCGAATGGGCAGGTAG 3'
**15**	5' AAGCGTTTGCGATGCTTCCTTC 3'
**16**	5' CGCCATGTAGTGTATTGACC 3'
**17**	5' CGCTTTCACTCTTCGAACAAACAC 3'
**18**	5' ACTATGCGGCATCAGAGCAG 3'
**19**	5’ CGAAGCTTTAAGCGGTGATTGAGCGT 3’
**20**	5’ CGGTCGACTGTTGATTGTAAAAGGA 3’
**21**	5’ CGCCCGGGATATTTGGTAAATGATAATC 3’
**22**	5’ CGAGATCTGCATACGTTCAGTGGTTGTT 3’
**23**	5’ AACATGCCCCACCCCTCCCC 3’
**24**	5’ GCTGCATCAGGTCGGAGACGC 3’
**25**	5’ AACATGCCCCACCCCTCCCC 3’
**26**	5’ GTGGTCGAATGGGCAGGTAG 3’
